# Deep learning-based cancer survival prognosis from RNA-seq data: approaches and evaluations

**DOI:** 10.1186/s12920-020-0686-1

**Published:** 2020-04-03

**Authors:** Zhi Huang, Travis S. Johnson, Zhi Han, Bryan Helm, Sha Cao, Chi Zhang, Paul Salama, Maher Rizkalla, Christina Y. Yu, Jun Cheng, Shunian Xiang, Xiaohui Zhan, Jie Zhang, Kun Huang

**Affiliations:** 1 School of Electrical and Computer Engineering, Purdue University, West Lafayette, IN 47907 USA; 2 Department of Medicine, Indiana University School of Medicine, Indianapolis, IN 46202 USA; 3 Department of Electrical and Computer Engineering, Indiana University - Purdue University Indianapolis, Indianapolis, IN 46202 USA; 4 Department of Biomedical Informatics, The Ohio State University, Columbus, OH 43210 USA; 5 Department of Medical and Molecular Genetics, Indiana University School of Medicine, Indianapolis, IN 46202 USA; 6 National-Regional Key Technology Engineering Laboratory for Medical Ultrasound, School of Biomedical Engineering, Health Science Center, Shenzhen University, Shenzhen, 518060 China; 7 Guangdong Key Laboratory for Biomedical Measurements and Ultrasound Imaging, School of Biomedical Engineering, Shenzhen University, Shenzhen, 518060 China

**Keywords:** Deep learning, Cancer prognosis, Survival analysis, Tumor mutation burden, Cox regression

## Abstract

**Background:**

Recent advances in kernel-based Deep Learning models have introduced a new era in medical research. Originally designed for pattern recognition and image processing, Deep Learning models are now applied to survival prognosis of cancer patients. Specifically, Deep Learning versions of the Cox proportional hazards models are trained with transcriptomic data to predict survival outcomes in cancer patients.

**Methods:**

In this study, a broad analysis was performed on TCGA cancers using a variety of Deep Learning-based models, including Cox-nnet, DeepSurv, and a method proposed by our group named AECOX (AutoEncoder with Cox regression network). Concordance index and *p*-value of the log-rank test are used to evaluate the model performances.

**Results:**

All models show competitive results across 12 cancer types. The last hidden layers of the Deep Learning approaches are lower dimensional representations of the input data that can be used for feature reduction and visualization. Furthermore, the prognosis performances reveal a negative correlation between model accuracy, overall survival time statistics, and tumor mutation burden (TMB), suggesting an association among overall survival time, TMB, and prognosis prediction accuracy.

**Conclusions:**

Deep Learning based algorithms demonstrate superior performances than traditional machine learning based models. The cancer prognosis results measured in concordance index are indistinguishable across models while are highly variable across cancers. These findings shedding some light into the relationships between patient characteristics and survival learnability on a pan-cancer level.

## Background

With the high prevalence of neural networks and Deep Learning-based algorithms in the Computational Biology, it is clear that the advantages of optimization in a highly non-linear space are welcomed improvements in biomedicine [1–7]. In Bioinformatics, significant effort has been committed to harnessing transcriptomic data for multiple analyses [7–13] especially cancer survival prognosis [14, 15]. Faraggi and Simon [16] was the first study to use clinical information to predict prostate cancer survival through an artificial neural network model. Mobadersany et al. [17] integrated histological features, Convolutional Neural Networks (CNN), and genomics data to predict cancer prognosis via Cox regression. Despite of various existed applications on survival analysis such as [14, 15], the use of Deep-Learning Cox models was pioneered by Ching et al. [18], who applied Cox regression with neural networks (Cox-nnet) to predict survival using transcriptomic data became prevalent. Similarly, Katzman et al. [19] used DeepSurv with multi-layer neural networks for survival prognosis and developed a personalized treatment recommendation system.

**Fig. 1:**
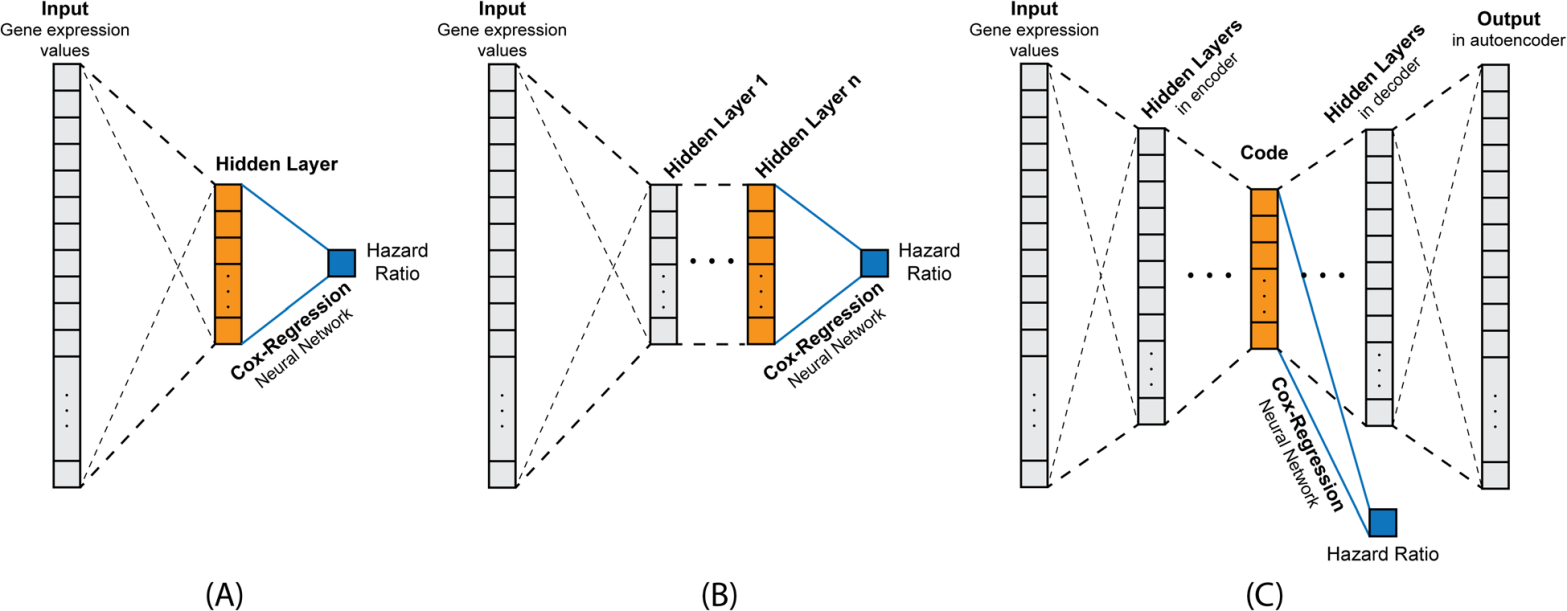
Neural network architectures of three Deep Learning-based models. **a** Cox-nnet with a single hidden layer; **b** DeepSurv with multiple hidden layers having consistent dimensions; **c** AECOX with multiple hidden layers in the both encoder and decoder part. Last hidden layers in all models were indicated in orange and were connect to a Cox regression neural networks with hazard ratios as the outputs

As a new and effective dimensionality reduction technique, the Autoencoder (AE) framework can lead to efficient lower dimensional representations using unsupervised or supervised learning [20–24]. In addition, Chaudhary et al. [25] also applied AE for dimensionality reduction and then used the low-dimensional representation of data to perform prognosis prediction using traditional method. In this paper, besides two recently developed Deep Learning based methods, namely Cox-nnet and DeepSurv, we also attempted an Autoencoder-based approach (called AECOX) for cancer prognosis prediction with simultaneous learning of lower dimensional representation of inputs. This approach is similar to Cox-nnet [18] and DeepSurv [19], as it implements neural networks with Cox regression, though the network architectures differ. In AECOX (Fig. 1c), the code from AE will link to a Cox regression layer for the prognosis. Both losses from the AE networks and Cox regression layer will be counted to train the entire network weights through back-propagation. AECOX is with symmetric structure of the Autoencoder, and can accept any number of hidden layers. We refer readers to the Additional file 1 for more detailed settings of AECOX.

To evaluate the prediction performance, we adopt two metrics, namely the concordance index and *p*-value of log-rank test. These metrics are used in comparing two state-of-the-art Deep Learning-based prognosis models (i.e., Cox-nnet, DeepSurv) with AECOX, in a pan-cancer study covering 12 TCGA (The Cancer Genome Atlas) cancers. In addition, we use Partitioning Around Medoids (PAM) clustering algorithm [26] on the last hidden layer for each model to evaluates how well the models discriminate subgroups in the lower dimensional space. *P*-value of log-rank test based on K groups of Kaplan-Meier survival curve is the metric used for evaluation [27].

As we compared the prognosis prediction performance across 12 cancer types, we wonder whether the performance is related to tumor mutation burden and overall survival time. Tumor mutation burden (TMB) is a measurement of mutations in tumor [28, 29] and is an important genomic marker that is closely associate with immunotherapy and survival prognosis [30–34]. While incorporating TMB feature into input does not increase the prediction performances, we found that TMB is negatively correlated with overall survival time statistics, and both of them are correlated with the concordance index for all three models across cancer types, suggesting an association between TMB, overall survival time, and disease prognosis accuracy.

Overall, we observed comparative results across three different Deep Learning-based cancer survival prognosis models in terms of concordance index. We also investigated the lower dimensional representation that conveyed by Deep Learning algorithms. By inspecting the relationship between TMB, overall survival statistics, and concordance index across 12 cancer types, we confirmed an association among them, suggesting a future study direction of patient stratification and integrative analysis.

## Method

### Integrating Cox proportional hazards model with neural networks

The neural network architectures of all three Deep Learning-based approaches are provided in Fig. 1. Cox-nnet (Fig. 1a) is the most succinct model with only one hidden layer, while DeepSurv (Fig. 1b) uses multiple hidden layers of consistent dimensions and treats the number of hidden layers as a hyper-parameter. Similarly, AECOX also treats the number of hidden layers as a hyper-parameter, but the hidden layers lay symmetrically in the encoder and decoder (Fig. 1c). All three models employed the same Cox proportional hazards model. However, Cox-nnet and DeepSurv accept the output of the last hidden layer to the Cox model while AECOX uses the low-dimensional code as the input. The output hazard ratio was then compared to the ground truth and the details of evaluation metrics are provided earlier. The reason we introduced AECOX is to explore the feasibility of simultaneously generating a low-dimensional representation of the data while developing an effective model for prognosis.

The Cox proportional hazards model, also known as the Cox model, was developed to models the age specific failure rate or hazard function [35] at time *t* for patient *i* with covariate vector *X*_*i*_ by.

1 $$ h\left(t|{X}_i\right)={h}_0(t)\exp \left(\sum \limits_{k=1}^K{\beta}_k{X}_{ik}\right) $$

The partial likelihood *L*_*i*_ for patient *i*, which is defined to be the probability of occurrence of a death event at time *Y*_*i*_ for patient *i*, is found to be

2 $$ {L}_i\left(\beta \right)=\frac{h\left({Y}_i|{X}_i\right)}{\sum_{j:{Y}_j\ge {Y}_i}h\left({Y}_i|{X}_j\right)}=\frac{h_0\left({Y}_i\right){\theta}_i}{\sum_{j:{Y}_j\ge {Y}_i}{h}_0\left({Y}_i\right){\theta}_j}=\frac{\theta_i}{\sum_{j:{Y}_j\ge {Y}_i}{\theta}_j} $$

at time *Y*_*i*_ for patient *i*. Where ${\theta}_i=\exp \left(\sum \limits_{k=1}^K{\beta}_k{X}_{ik}\right)$. *β* = (*β*_1_, *β*_2_, …, *β*_*K*_) are the *K* parameters to be estimated. The summation in denominator is carried out over all patients *j* (including patient *i*) for which a death event did not occur before time *Y*_*i*_. The partial likelihood for all patients is then defined as

3 $$ L\left(\beta \right)=\prod \limits_{i:{C}_i=1}{L}_i\left(\beta \right) $$

where *C*_*i*_ = 1 indicates the occurrence of a death event. The log partial likelihood of Cox model is then obtained as

4 $$ \ell \left(\beta \right)=\sum \limits_{i:{C}_i=1}\left(\sum \limits_{k=1}^K{\beta}_k{X}_{ik}-\log \left({\sum}_{j:{Y}_j\ge {Y}_i}{\theta}_j\right)\right) $$

Values of the parameters *β* = (*β*_1_, *β*_2_, …, *β*_*K*_) are then obtained through maximum likelihood estimation (MLE), that is

5 $$ \hat{\beta}={\mathrm{argmax}}_{\beta}\left(\ell \left(\beta \right)\right) $$

Alternatively, since the Cox model utilizes a regression model that can be implemented as neural network with weights *β* = (*β*_1_, *β*_2_, …, *β*_*K*_), values of these weights were obtained through back-propagation. This approach was embedded in all the aforementioned models and was denoted by the blue line with caption “Cox-Regression Neural Network” in Fig. 1.

These models offer several advanced features: (1) a highly non-linear function is learned, (2) neural networks and Cox proportional hazards regression are integrated together enabling the entire weights of the models to be learned through back-propagation, (3) the number of hidden layers and hidden layer dimensions were treated as hyper-parameters that can be fine-tuned, and (4) dimensionality reduction in conjunction with supervised learning is achieved.

To demonstrate the advantages of Deep Learning-based prognosis models, we also compared three traditional machine learning based models for prognosis, they are: Cox proportional hazards model with R package “glmnet” [36], Random Survival Forest (RSF) [37], and Support Vector Machine (SVM) [25]. Particularly, in Chaudhary et al. [25], we implemented their SVM model according to the top 100 mRNA-seq features selected from ANOVA (Analysis of variance) [38].

### Regularization, loss functions and hyper-parameters

Despite the fact that the aforementioned Deep Learning-based approaches shared the same Cox regression network and used the hazard ratio as the output (Table 1), yet certain differences existed among the models. Currently all three models used the L2 norm regularization in the final learning after hyper-parameters tuning as it gave the optimal validation accuracy. While all models attempted Dropout and the L2 norm regularization (Ridge Regularization [39]) to penalize the network weights, AECOX also included L1 norm regularization (Least Absolute Shrinkage and Selection Operator, LASSO in short [40]) and elastic net [41].

**Table 1:** Comparison of model architectures and settings across three Deep Learning-based cancer survival prognosis approaches

Properties	Models		
	Cox-nnet	DeepSurv	AECOX
Deep Learning Architecture	Single-layer neural networks	Multi-layer neural networks	Multi-layer Autoencoder neural networks
Deep Learning Programming Framework	Theano	Theano, Lasagne	PyTorch
Hyper-parameters	L2 regularization weight *λ*.	Learning rate; Number of hidden layers; Hidden layer sizes; Learning rate decay; Momentum; L2 regularization weight *λ*; Dropout rate.	Learning rate; Autoencoder input-output error weight *λ*_1_; L1 regularization weight *λ*_2_; L2 regularization weight *λ*_3_; Dropout rate; Number of hidden layers; Regularization method.
Hyper-parameters Searching Methods	Line search	Sobol solver	Sobol solver
Number of iterations for searching hyper-parameters	12	100	100
Maximum epochs	4000	500	300
Number of Hidden Layers	1	1, 2, 3, or 4	0, 2, 4, 6, or 8
Last hidden Layer sizes	Integer value in range [131, 135]	Integer value in range [30, 50]	16
Regularization Methods	L1, L2, Dropout	L2, Dropout	Dropout, L1, L2, Elastic Net
Basic Objective (Loss) Functions	$\hat{\Theta}={\mathrm{argmin}}_{\Theta}\left\{{\sum}_{i:{C}_i=1}\left(\sum \limits_{k=1}^K{\beta}_k{X}_{ik}-\log \left({\sum}_{j:{Y}_j\ge {Y}_i}{\theta}_j\right)\right)\right\}$		
Optimization Methods	Nesterov accelerated gradient descent	Stochastic gradient descent (SGD)	Adaptive Moment Estimation (Adam)
Network Architectures	(Input Layer) – (Hidden Layer) (tanh) – (Hazard Ratio)	(Input Layer) – (Hidden Layer) (ReLU/SELU) – … – (Hidden Layer) (ReLU/SELU) – (Hazard Ratio)	(Input Layer) – (Hidden Layers) (ReLU/Dropout) – (Code) – (Hidden Layers) (ReLU/Dropout) – (Output Layer); (Code) (tanh) – (Hazard Ratio)

The structure of loss functions among models shared a common base formula (Table 1), but each approach used additional penalization. Specifically, both Cox-nnet and DeepSurv used the same objective (loss) function:

6 $$ \hat{\Theta}={\mathrm{argmin}}_{\Theta}\left\{{\sum}_{i:{C}_i=1}\left(\sum \limits_{k=1}^K{\beta}_k{X}_{ik}-\log \left({\sum}_{j:{Y}_j\ge {Y}_i}{\theta}_j\right)\right)+\lambda {\left\Vert \Theta \right\Vert}_2^2\right\} $$

whereas AECOX took into account the Autoencoder’s input-output difference:

7 $$ \hat{\Theta}={\mathrm{argmin}}_{\Theta}\left\{{\lambda}_1\mathrm{MSE}\left({X}_{input},{X}_{output}\right)\right.+\left(1-{\lambda}_1\right){\sum}_{i:{C}_i=1}\left(\sum \limits_{k=1}^K{\beta}_k{X}_{ik}-\log \left({\sum}_{j:{Y}_j\ge {Y}_i}{\theta}_j\right)\right)+\left.{\lambda}_2{\left\Vert \Theta \right\Vert}_1+{\lambda}_3{\left\Vert \Theta \right\Vert}_2^2\right\} $$

Here Θ denotes to the neural networks’ weights to be learned, including hidden layer weights and Cox regression neural network weights, *X*_*input*_ and *X*_*output*_ are the input and output covariate vectors of Autoencoder, respectively. MSE(∙) is the mean squared error function. The hyper-parameter *λ*_1_ balances the loss between Autoencoder’s input-output difference which is a measure of dimensionality reduction and the Cox hazard, which is a measure of regression based supervised learning. The combination of *λ*_2_ and *λ*_3_ permits the utilization of Elastic Net regularization. Forcing *λ*_2_ = 0 results in L2 regularization, whereas forcing *λ*_3_ = 0 results in L1 regularization. To optimize the objective functions given above, Cox-nnet, DeepSurv, and AECOX use Nesterov accelerated gradient descent [42], stochastic gradient descent (SGD) [43], and adaptive moment estimation (Adam) optimizer [44], respectively. AECOX adopted Adam optimizer as it is more computationally efficient and require little tuning on hyper-parameters.

As shown in Table 1, Cox-nnet has one hyper-parameter to be fine-tuned, and thus a linear search technique was adopted, whereas DeepSurv and AECOX had multiple hyper-parameters in a high dimensional space. It is thus unrealistic to perform a linear search in each dimension of the hyper-parameter space as the computational complexity would be *O*(*n*^*p*^) for *p* hyper-parameters. Instead, DeepSurv and AECOX utilize the Sobol solver [45] in the Optunity python package [46]. Given a search time *q* (e.g., *q* = 100), the Sobol solver samples *q* points assuming the hyper-parameters are uniformly distributed in *p*-dimensional space. This reduces the computational complexity to *O*(*nq*), regardless of how large the value of *p* is.

### Data preprocessing and statistics

Genes with lowest 20% absolute expression values and lowest 10% variance across samples were removed. This denoising step was performed via the TSUNAMI package (https://apps.medgen.iupui.edu/rsc/tsunami/) [15], ensuring model robustness and reducing irrelevant noise.

The expression data were then rescaled with natural logarithm operation:

8 $$ {X}_{input}=\log \left({X}_{original}+1\right) $$

where *X*_*original*_ was the original non-negative RNA sequencing expression values (Illumina Hi-Seq RNA-seq v2 RSEM normalized), and *X*_*input*_ was the input covariate vector for the models. Subsequently each gene expression at row *r* in the input data was normalized as

9 $$ {X}_{input}^{(r)}=\frac{X_{input}^{(r)}-\min \left({X}_{input}^{(r)}\right)}{\max \left({X}_{input}^{(r)}\right)-\min \left({X}_{input}^{(r)}\right)\ } $$

This step ensured that each row of the gene expression contributed to the model on an equal scale.

Table 2 provides a summary of the median and range in terms of age and survival months for the TCGA data. Each dataset was split into training, validation, and testing sets in a proportion of 60, 20, and 20% respectively. Confounding effects [47] were minimized by randomly shuffling the data 1000 times and choosing the 5 pairs of training/validation/testing sets with lowest corresponding differences. The differences that were minimized is the summation of (1) standard deviation of male/female ratio on training/validation/testing sets, (2) standard deviation of overall survival time’s standard deviation on training/validation/testing sets, (3) standard deviation of overall survival time’s mean on training/validation/testing sets, (4) standard deviation of the ratio of deceased group to whole population on training/validation/testing sets, and (5) standard deviation of the ratio of tumor stages to whole population on training/validation/testing sets. Thus, survival prognosis was estimated for each cancer type 5 times.

**Table 2:** The Cancer Genome Atlas (TCGA) 12 cancers’ statistics. Cancers were sorted based on averaged concordance index in descending order according to Fig. 2

TCGA Cancers	TCGA Cancer Abbreviations	Total Cases	Censored (Living) Group	Uncensored (Deceased) Group	Number of Genes After Pre-processing	Age		Overall Survival Months	
						Median	Range	Median	Range
Kidney	KIRP	286	242	44	17,867	61.5	28–88	25.45	0.00–194.65
Kidney	KIRC	531	357	174	17,870	61	26–90	38.96	0.00–149.05
Liver	LIHC	369	239	130	17,963	61	16–90	19.32	0.00–120.73
Breast	BRCA	1083	933	150	18,030	58	26–90	27.56	0.00–282.69
Cervical	CESC	302	231	71	17,731	46	20–88	20.93	0.00–210.51
Lung	LUAD	495	315	180	17,715	66	38–88	21.55	0.00–238.11
Bladder	BLCA	402	225	177	18,008	69	34–90	17.61	0.43–165.90
Head-Neck	HNSC	514	296	218	17,968	61	19–90	21.46	0.07–210.81
Pancreatic	PAAD	176	83	93	17,150	65	35–88	15.20	0.00–90.05
Ovarian	OV	299	119	180	17,635	58	30–87	31.27	0.30–180.06
Stomach	STAD	397	244	153	18,172	67	30–90	14.03	0.00–122.21
Lung	LUSC	489	283	206	18,030	68	39–90	21.91	0.00–173.69

In this study, TCGA mutation annotation files (MAFs), containing subsets of the patients for prognosis tasks, were used to calculate TMB summary statistics, including mean, median, max, and 20, 10, 5% tail cut values. These characteristics were used for examining correlation between TMB and concordance index.

### Evaluation metrics

We evaluated model performance with concordance index and the *p*-value of log-rank test. Concordance index had been widely used for evaluating survival prognosis models [48–50]. Its value ranges from 0 to 1 and it describes how well models differentiated groups (censored and uncensored groups, or living and deceased groups) [50–53]. A concordance index of 0.5 indicates that a model was ineffective and is viewed to have generated a random prediction with respect to ground truth. Values above 0.5 indicate improved prediction by a model, with increased performance being conveyed by a concordance index approaching 1. Values below 0.5 indicate that a model predicted values that are the opposite of the ground truth. Higher concordance index values indicate better capability of model to perform cancer survival prognosis.

*P*-values were derived by dichotomizing the hazard ratios through median value and performing log-rank tests [54–56] between the resulted high-risk and low-risk groups. Model performance was then assessed wherein a lower *p*-value represents an enhanced ability to distinguish two patient groups.

To evaluate the performances across cancer types and across model types, two-way ANOVA [38] is adopted. Pairwise paired t-test [57, 58] and the linear mixed-effects models test from the R package “nlme” [59, 60] are also used. The linear mixed-effects models test is to test between pairs of models while accounting for random effects. The mixed effect model assumed the data (performances) to be dependent within each cancer type and independent across cancer types.

## Results

The performance comparison was conducted at pan-cancer level using 12 cancer from The Cancer Genome Atlas (TCGA). These 12 cancers were chosen due to their relatively large sample sizes and sufficient information about patient outcomes. The specific cancers analyzed in this paper were (1) Urothelial Bladder Carcinoma (BLCA); (2) Breast Invasive Carcinoma (BRCA); (3) Cervical Squamous Cell Carcinoma and Endocervical Adenocarcinoma (CESC); (4) Head-Neck Squamous Cell Carcinoma (HNSC); (5) Kidney Renal Clear Cell Carcinoma (KIRC); (6) Kidney Renal Papillary Cell Carcinoma (KIRP); (7) Liver Hepatocellular Carcinoma (LIHC); (8) Lung Adenocarcinoma (LUAD); (9) Lung Squamous Cell Carcinoma (LUSC); (10) Ovarian Cancer (OV); (11) Pancreatic Adenocarcinoma (PAAD); and (12) Stomach Adenocarcinoma (STAD). In this paper, we used the expression data of Illumina Hi-Seq RNA-seq v2 RSEM normalized genes from TCGA.

### Performance comparison

**Fig. 2:**
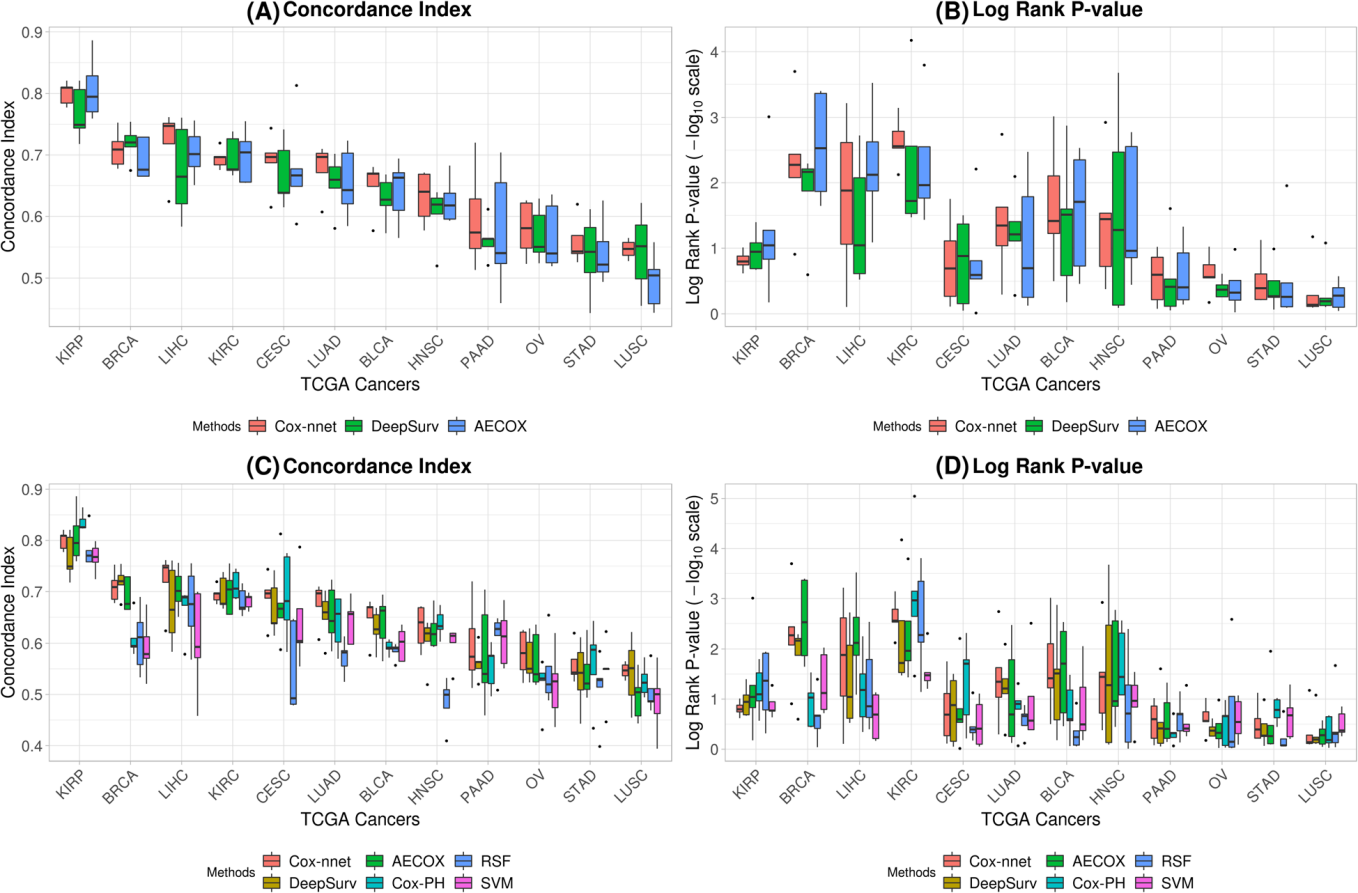
**a**, **b**: Performance comparisons between three Deep Learning-based models across 12 TCGA (The Cancer Genome Atlas) cancers. **a** concordance index; **b** *p*-value of log-rank test (in −log_10_ scale). **c**, **d**: Performance comparisons between three Deep Learning-based models and three traditional machine learning models across 12 TCGA (The Cancer Genome Atlas) cancers. **c** concordance index; **d** *p*-value of log-rank test (in −log_10_ scale). Cancers were sorted based on averaged concordance index across models and experiments. For detailed cancer names, please refer to the Additional file 1

Figure 2a and b present concordance indices and *p*-values of log-rank tests among the different models and different cancer datasets, wherein the cancers in x-axis were sorted based on the averaged concordance index values among all models and experiments. It is observed that models for cancers like KIRP, BRCA, and LIHC yield median concordance indices of at least 0.7, whereas some cancers like STAD and LUSC yield median concordance indices of approximately 0.5. This led to our further investigation with tumor mutation burden (TMB) and overall survival time as described earlier. We also made a comparison between three traditional machine learning models (Fig. 2c, d). Specifically, we presented the results in Fig. 2 as two parts in order to directly visualize the comparison between Deep Learning-based models and traditional machine learning based models in Fig. 2c and d.

Since five experiments were carried out for each cancer type and each model type, we compared the performances (via concordance index and *p*-value of log-rank test) for all 12 TCGA cancer types using pairwise paired t-test among all models (Table 3a) and the linear mixed-effects models test (Table 3b). In this case we considered a model to be better than another if a higher concordance index or a lower *p*-value of log-rank test was observed. Thus, a positive t-statistic in Table 3a or a positive coefficient in Table 3b was used to conclude that the model (distribution 1) was better than the other (distribution 2) with respect to the concordance index. In the case of the p-value of log-rank test, a negative t-statistic or coefficient was used to reach the same inference.

**Table 3:** Model-wised performances comparison at pan-cancer level (12 TCGA (The Cancer Genome Atlas) cancer types) by pairwise paired t-test (A) and linear mixed-effects models test (B), according to metrics concordance index and p-value of log-rank test. Note that for concordance index, larger t-statistic/coefficient indicated better performance at pan-cancer level, while the *p*-value of log-rank test was on the contrary

(A) Pairwise Paired T-test						
			Distribution 2			
			DeepSurv		AECOX	
			t	P	t	P
Distribution 1	Cox-nnet	concordance index	3.1843	2.32E-03	3.2281	2.04E-03
		p-value of log-rank test	−1.4006	1.67E-01	−0.8962	3.74E-01
	DeepSurv	concordance index	–	–	−0.6732	5.03E-01
		p-value of log-rank test	–	–	0.5164	6.07E-01
Notes: t denotes the pairwise paired Student’s t-test statistic, P denotes the p-value obtained.						
(B) Linear Mixed-Effects Models Test						
			Distribution 2			
			DeepSurv		AECOX	
			*β*	P	*β*	P
Distribution 1	Cox-nnet	concordance index	0.0195	1.97E-02	0.0142	1.12E-01
		p-value of log-rank test	−0.0489	2.52E-01	−0.0294	4.85E-01
	DeepSurv	concordance index	–	–	−0.0052	5.85E-01
		p-value of log-rank test	–	–	0.0195	6.62E-01

Notes: *β* denotes the coefficient (slope) of linear mixed-effects models, P denotes the p-value obtained.

As can be observed from Table 3b, all models have a similar performance since most of the test results of their linear mixed-effects models are insignificant. Both Table 3a and Table 3b concluded that among Deep Learning-based approaches, Cox-nnet provided the overall optimal survival prognosis results at pan-cancer level, with respect to the concordance index and the p-value of log-rank test. This advantage of Cox-nnet is due to a simpler neural network architecture and reduced search space for hyper-parameters. Additional file 1: Table S10-S11 presented the same quantitative comparison of performances for Deep Learning-based and traditional machine learning models. All three Deep Learning models demonstrated superior performance than traditional machine learning models, suggesting the advantages of Deep Learning approaches on prognosis prediction.

### Lower dimensional representation

The final hidden layer (or the code in AECOX), highlighted in orange in Fig. 1, produces a lower dimensional representation of the input and is one of the intrinsic properties in Deep Learning-based algorithms [18, 21, 61, 62]. By using the Partitioning Around Medoids (PAM) clustering algorithm [26] on the output of the last hidden layer after the network is trained, we can then inspect the original covariate vector in a lower dimensional space. The most suitable number of clusters (ranging from 2 to 10) was determined by maximizing the averaged silhouette score [63, 64]. As depicted in Table 4, Cox-nnet appeared to have overall better *p*-values of the log-rank test measured between different clusters, indicating a better capacity of dimensionality reduction for 9 cancers (KIRP, KIRC, LIHC, BRCA, CESC, LUAD, HNSC, OV, LUSC).

**Table 4:** P-value of the log-rank test of lower dimensional representation, generated by Partitioning Around Medoids (PAM) clustering algorithm on the last hidden layers of three Deep Learning-based approaches (testing set only). 12 TCGA (The Cancer Genome Atlas) cancers are being compared. Bolded values indicate the smallest *p*-value among three Deep Learning approaches, refer to better low dimensional representation

TCGA Cancers	*P*-values of log-rank test by models		
	Cox-nnet	DeepSurv	AECOX
KIRP	**7.71E-02**	2.45E-01	1.40E-01
KIRC	**2.38E-03**	9.79E-02	3.01E-01
LIHC	**3.57E-01**	6.14E-01	3.86E-01
BRCA	**4.45E-01**	4.81E-01	4.85E-01
CESC	**2.45E-01**	3.26E-01	3.92E-01
LUAD	**9.58E-02**	4.07E-01	1.11E-01
BLCA	3.27E-01	**2.67E-01**	4.94E-01
HNSC	**3.38E-01**	4.06E-01	6.19E-01
PAAD	2.97E-01	4.04E-01	**2.29E-01**
OV	**2.80E-01**	3.38E-01	4.42E-01
STAD	5.72E-01	**2.67E-01**	7.01E-01
LUSC	**3.10E-01**	4.14E-01	6.05E-01

The boldface *p*-value indicates it is the smallest one among all three algorithms

### Relationship between prognosis prediction performances and tumor mutation burden

From the performances within a cancer type across models in Fig. 2 and results in Table 3, it appeared that all models achieve respectable performances measured by concordance index. We also found that performance (concordance index) was more significantly associated with cancer types than algorithms (two-way ANOVA: Cancer type p-value <2E-16, Model type *p*-value = 9.57E-02). This observation suggests that intrinsic characteristics of different cancer types have a large influence on the performance of prognosis models. One such characteristics is the tumor mutation burden (TMB), which is known to vary largely between different types of cancers.

**Fig. 3:**
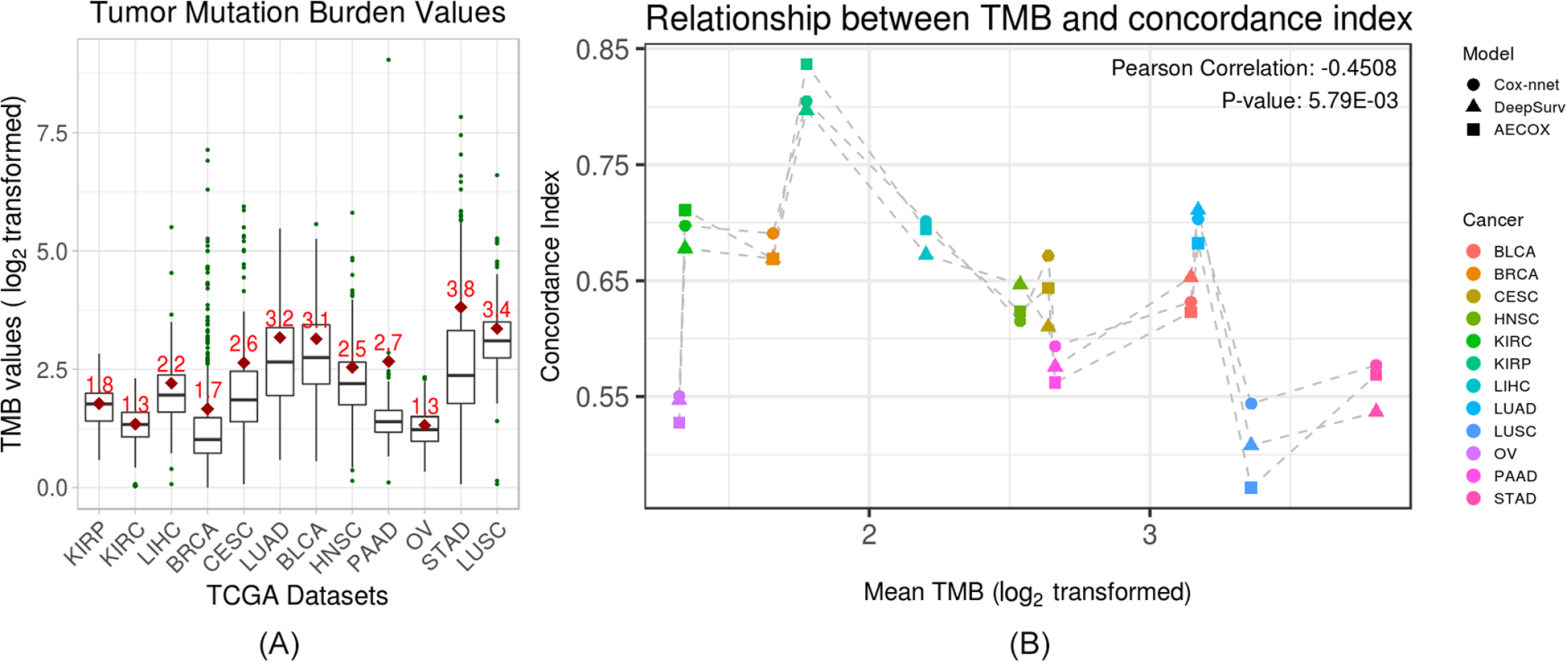
Cox-nnet performances with TMB feature input and without TMB feature input across 12 TCGA (The Cancer Genome Atlas) cancers. **a** concordance index; **b** *p*-value of log-rank test (in −log_10_ scale). Red diamonds and texts on the boxplot indicate the mean values. Cancers were ordered based on Fig. 2. Note that the performances are differ from Fig. 2 due to new patient cohorts (intersection of patients who has both RNA-seq data and TMB data). For detailed cancer names, please refer to the Additional file 1

TMB was increasingly used as a marker in predicting efficacy of immunotherapy [33] and was also shown to be a predictor of prognosis [34]. Since the ability to train a cancer survival prognosis model across cancer types varies significantly, we explored whether TMB can be associated with these changes. By inspecting the mutation information associated with different cancer types, we observed that the performance of survival prognosis models was associated with tumor mutation burden (TMB) characteristics. Specifically, we observed that all TMB characteristics were negatively correlated with concordance index especially the mean TMB (Mean TMB: Pearson *ρ* = − 0.45 (Fig. 3b); Median TMB: Pearson *ρ* = − 0.30; Maximum TMB: Pearson *ρ* = − 0.40; 20% tail TMB: Pearson *ρ* = − 0.32; 10% tail TMB: Pearson *ρ* = − 0.32; 5% tail TMB: Pearson *ρ* = − 0.30).

**Fig. 4:**
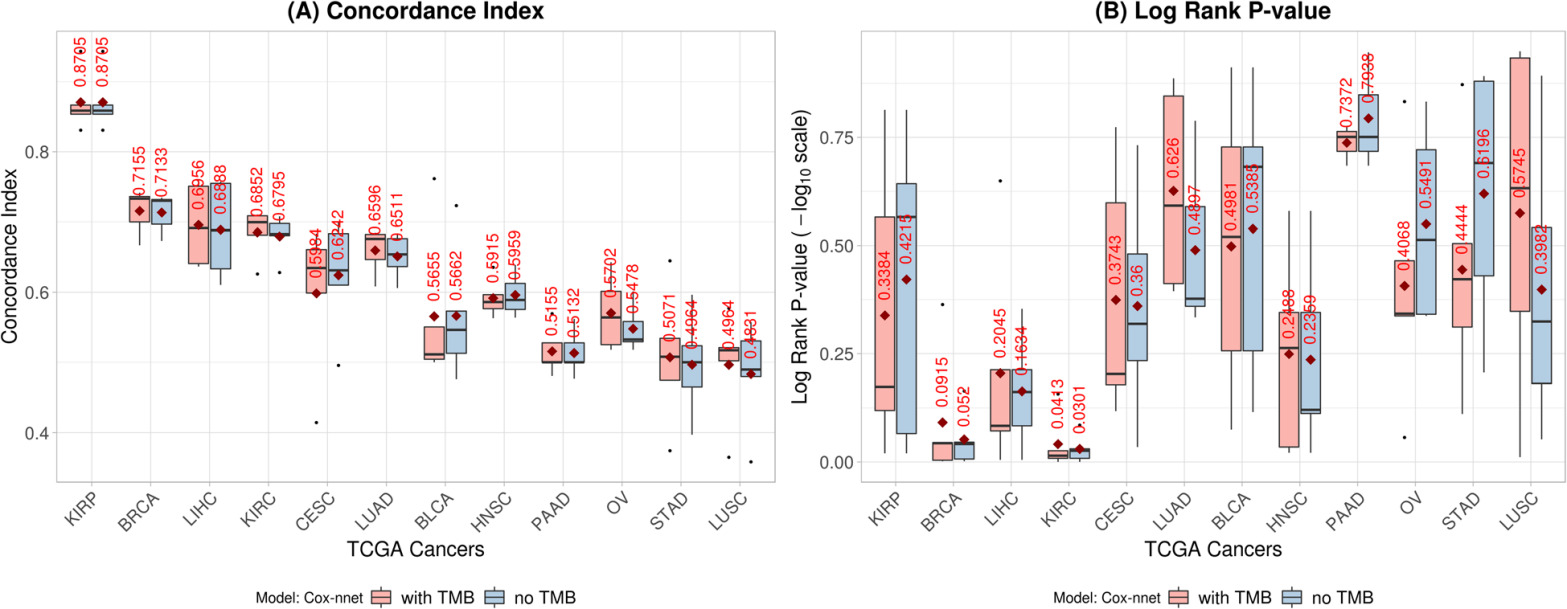
**a** Box plot of log_2_ transformed tumor mutation burden (TMB) values from all available TCGA (The Cancer Genome Atlas) patients with respect to each cancer type, ordered according to Fig. 2. Texts and diamond symbols in red color indicated the mean values. **b** Mean TMB versus averaged concordance index results across 12 cancer types with three survival prognosis models. Pearson *ρ* =  − 0.45 (*p*-value = 5.79E-03). Individual model correlations are range from −0.46 to −0.44, described in Additional file 1: Table S4. Other results of TMB statistics versus concordance index were shown in Additional file 1: Figure S2 – Figure S6.

One interesting question is then if the incorporating TMB in the model would enhance the model performance. To investigate this, we take the joint subset of patients who have both RNA-seq data and TMB data, performed survival prognosis with Cox-nnet model (the method which has the best performance) with and without TMB feature, respectively. As shown in Fig. 4, although there is a slight improvement on concordance index (average value = 0.003419) after TMB feature is incorporated, the correlation between improved concordance index (mean) and mean TMB values is 0.0688 across 12 TCGA cancers, suggesting that introducing TMB feature into a mRNA-seq based learning model does not substantially improve the performance for Cox-nnet.

Next, we found the correlation between the mean of overall survival times and the mean of TMB values is − 0.6853 (Pearson) and − 0.7133 (Spearman) across 12 cancers, and the correlation between the variance of overall survival times and the variance of TMB values is − 0.6159 (Pearson) and − 0.2448 (Spearman), suggesting a strong correlation between higher TMB and shorter overall survival times statistics. Where the correlation between the mean of overall survival times and the mean of concordance index is 0.4271 (Pearson) and 0.4126 (Spearman).

## Discussion

Overall our study demonstrated that the Deep Learning architecture can be effectively applied for cancer prognosis prediction with Cox-proportional hazard model incorporated. We found that Deep Learning-based model demonstrated superior performances comparing to traditional machine learning models. Among the three Deep Learning-based models tested, we observed that Cox-nnet, which has the most succinct neural network structure, resulted in better prognosis performances in the measurement of concordance index and *p*-value of log-rank test. We showed that integrating autoencoder with Cox regression network does not significantly improve the prognosis performances. These results highlight an important issue in Deep Learning approaches—namely simpler models often perform similar or better to more complex models in biological data.

From the associated fine-tuned hyper-parameters (Additional file 1: Table S5-S9) during the hyper-parameters tuning (with optimal validation accuracy), we found that Deep Learning-based algorithms and traditional machine learning based algorithms, especially with multiple hyper-parameters, tends to converge into different local minima with different hyper-parameter values. For example, the optimal parameter pairs of AECOX are not consistent in five different folds even when these experiments are from same cancer (e.g.*,* TCGA BRCA cancer). This result can potentially be due to the curse of dimensionality [65]: with limited number of different training samples and large number of parameters (e.g.*,* the hidden layer weights in Deep Learning-based models), the optimization may not guarantee to converge to same local minima. These observations lead us to rethink the robustness of training procedure – especially when higher performances are observed on Cox-nnet where it has the least hyper-parameter tuning effort.

We also noticed a negative correlation between TMB values and prognosis prediction performances. The relationship between TMB and prognosis have been examined in existing literatures in cancer biology for individual cancer types. For example, Owada-Ozaki et al. [66] examined the relationship between individual TMB and prognosis and concluded that high TMB is a poor prognostic factor in non-small cell lung cancer (NSCLC). A similar pattern occurs between TMB and prognosis specifically for lung adenocarcinomas (a subtype of NSCLC) (Naidoo et al. [67]). Our pan-cancer analyses are agreeing with these findings yet have a different conclusion. We observed that TMB is correlated with prognosis performances (concordance index), however, integrating TMB to the Cox-nnet model does not improve the performances at the pan-cancer level. By further examining the relationships behind these features and results, we found that TMB is highly correlated with overall survival times (both in mean and variance) across cancer types. Specifically, lower TMB value is associated with longer mean overall survival time, concluded that TMB is a marker for tumor malignancy. These findings lead us to speculate that TMB either affect or are affected by overall survival time, but may not directly contribute to prognosis prediction when gene expression data are used. However, with a strong correlation to TMB, shorter overall survival times leads to worse prognosis performance, suggesting a direct relationship between overall survival statistics and prognosis performances. These findings will guide us to design future experiments to further explain the detailed relationships especially the dependency among TMB, survival times, and prognosis performances at pan-cancer level.

## Conclusion

Bringing artificial intelligence into clinical and cancer studies [6, 68–70] can unravel numerous interpretabilities behind the data. In this paper, we focused on three different Deep Learning-based cancer prognosis models. The survival predictions are conducted across 12 TCGA cancer types with sufficient number of patients and survival information. We found that Deep Learning based algorithms demonstrate superior performances than traditional machine learning based models. We also found that the cancer prognosis results measured in concordance index are indistinguishable across models while are highly variable across cancers by two-way ANOVA. The highest concordance index that models can predict is renal papillary cell carcinoma (KIRP), while the lowest concordance index is observed for lung squamous cell carcinoma (LUSC). We then examined the relationships between TMB statistics, overall survival statistics, and concordance indices across 12 cancers. We found that although TMB and overall survival times are negatively correlated with concordance indices across the cancer types, integrating TMB does not improve the prognosis prediction performance for individual cancers significantly, whereas TMB has a strong correlation with overall survival times. These findings will guide us to explore the relationships between patient characteristics and survival learnability in a pan-cancer level in the future work.

## Additional file

**Additional file 1: Figure S1.** An example framework of the AECOX model with four hidden layers. **Table S1.** The network design of AECOX. **Table S2.** The hyper-parameters of AECOX to be searched. **Table S3.** Performances of testing set in TCGA Kidney Renal Clear Cell Carcinoma (KIRC) dataset. Bolded texts indicated optimal results among all models. **Table S4.** Individual model correlations (Pearson *ρ*) of mean TMB (Fig. 2). **Figure S2.** Relationship between concordance index and median TMB. Pearson *ρ* =  − 0.30 (*p*-value = 7.75E-02). **Figure S3.** Relationship between concordance index and max TMB. Pearson *ρ* =  − 0.40 (*p*-value = 1.68E-02). **Figure S4.** Relationship between concordance index and 20% tail TMB. Pearson *ρ* =  − 0.32 (p-value = 5.51E-02). **Figure S5.** Relationship between concordance index and 10% tail TMB. Pearson *ρ* =  − 0.32 (*p*-value = 5.93E-02). **Figure S6.** Relationship between concordance index and 5% tail TMB. Pearson *ρ* =  − 0.30 (*p*-value = 7.45E-02). **Table S5.** Fine-tuned hyper-parameters of Cox-nnet (L2 penalty weight *λ*) across 12 cancer types and 5 experiments (folds). **Table S6.** Fine-tuned hyper-parameters of DeepSurv across 12 cancer types and 5 experiments (folds). **Table S7.** Fine-tuned hyper-parameters of AECOX across 12 cancer types and 5 experiments (folds). Note that we fixed *λ*_2_ = 0 to only impose L2 sparsity. **Table S8.** Fine-tuned hyper-parameters of Random Survival Forest (RSF) (number of the trees) across 12 cancer types and 5 experiments (folds). **Table S9.** Fine-tuned hyper-parameters of SVM (*α*, weight of penalizing the squared hinge loss in the objective function) across 12 cancer types and 5 experiments (folds). **Table S10.** Model-wised performances comparison at pan-cancer level (12 TCGA (The Cancer Genome Atlas) cancer types) by pairwise paired t-test, according to metrics concordance index and p-value of log-rank test. Note that for concordance index, larger t-statistic/coefficient indicated better performance at pan-cancer level, while the p-value of log-rank test was on the contrary. **Table S11.** Model-wised performances comparison at pan-cancer level (12 TCGA (The Cancer Genome Atlas) cancer types) by linear mixed-effects models test, according to metrics concordance index and p-value of log-rank test. Note that for concordance index, larger t-statistic/coefficient indicated better performance at pan-cancer level, while the p-value of log-rank test was on the contrary.

## Data Availability

The results shown here are in whole or part based upon data generated by the TCGA Research Network: https://www.cancer.gov/tcga. All mRNA-seq data were based on illuminahiseq_rnaseqv2-RSEM_genes_normalized from Broad GDAC Firehose (https://gdac.broadinstitute.org/) transcriptomic data as the inputs to the models.

## Abbreviations

ANOVA: Analysis of variance
MLE: Maximum likelihood estimation
PAM: Partitioning around medoids
TCGA: The Cancer Genome Atlas
TMB: Tumor mutation burden
TSUNAMI: Translational Bioinformatics Tool Suite For Network Analysis And Mining

## References

[1] LeCun Y, Bengio Y, Hinton G (2015). Deep learning. Nature.

[2] Min S, Lee B, Yoon S (2017). Deep learning in bioinformatics. Brief Bioinform.

[3] Leung MK, Xiong HY, Lee LJ, Frey BJ (2014). Deep learning of the tissue-regulated splicing code. Bioinformatics.

[4] Chen Y, Li Y, Narayan R, Subramanian A, Xie X (2016). Gene expression inference with deep learning. Bioinformatics.

[5] Alipanahi B, Delong A, Weirauch MT, Frey BJ (2015). Predicting the sequence specificities of DNA- and RNA-binding proteins by deep learning. Nat Biotechnol.

[6] Huang Z, Zhan XH, Xiang SN, Johnson TS, Helm B, Yu CY, Zhang J, Salama P, Rizkalla M, Han Z, et al. SALMON: survival analysis learning with multi-Omics neural networks on breast Cancer. Front Genet. 2019;10. 10.3389/fgene.2019.00166 PMC6419526 30906311

[7] Johnson TS, Li SH, Franz E, Huang Z, Li SYD, Campbell MJ, Huang K, Zhang Y. PseudoFuN: Deriving functional potentials of pseudogenes from integrative relationships with genes and microRNAs across 32 cancers. Gigascience. 2019;8(5),giz046:1-13. 10.1093/gigascience/giz046 PMC6486473 31029062

[8] Yu CY, Xiang S, Huang Z, Johnson TS, Zhan X, Han Z, Abu Zaid MI, Huang K (2019). Gene Co-expression Network and Copy Number Variation Analyses Identify Transcription Factors Involved in Multiple Myeloma Progression. Front Genet.

[9] Feng C, Huang H, Huang S, Zhai YZ, Dong J, Chen L, Huang Z, Zhou X, Li B, Wang LL (2018). Identification of potential key genes associated with severe pneumonia using mRNA-seq. Exp Ther Med.

[10] Huang S, Feng C, Chen L, Huang Z, Zhou X, Li B, Wang LL, Chen W, Lv FQ, Li TS (2017). Molecular mechanisms of mild and severe pneumonia: insights from RNA sequencing. Med Sci Monit.

[11] Xiang S, Huang Z, Wang T, Han Z, Yu CY, Ni D, Huang K, Zhang J. Condition-specific gene co-expression network mining identifies key pathways and regulators in the brain tissue of Alzheimer's disease patients. BMC Med Genet. 2018;11(Suppl 6):115. 10.1186/s12920-018-0431-1 PMC6311927 30598117

[12] Zhan XH, Cheng J, Huang Z, Han Z, Helm B, Liu XW, Zhang J, Wang TF, Ni D, Huang K (2019). Correlation analysis of histopathology and Proteogenomics data for breast Cancer. Mol Cell Proteomics.

[13] Helm BR, Zhan X, Pandya PH, Murray ME, Pollok KE, Renbarger JL, Ferguson MJ, Han Z, Ni D, Zhang J, et al. Gene Co-Expression Networks Restructured Gene Fusion in Rhabdomyosarcoma Cancers. Genes-Basel. 2019;10(9):665. 10.3390/genes10090665 PMC6770752 31480361

[14] Huang S, Yang H, Li Y, Feng C, Gao L, G-f C, H-h G, Huang Z, Y-h L, Yu L (2016). Prognostic significance of mixed-lineage leukemia (MLL) gene detected by real-time fluorescence quantitative PCR assay in acute myeloid leukemia. Med Sci Monit.

[15] Shao W, Wang T, Huang Z, Cheng J, Han Z, Zhang D, Huang K. Diagnosis-Guided Multi-modal Feature Selection for Prognosis Prediction of Lung Squamous Cell Carcinoma. In: International Conference on Medical Image Computing and Computer-Assisted Intervention: 13-17 October 2019. Shenzhen: Springer; 2019. p. 113–21.

[16] Faraggi D, Simon R (1995). A neural-network model for survival-data. Stat Med.

[17] Mobadersany P, Yousefi S, Amgad M, Gutman DA, Barnholtz-Sloan JS, Vega JEV, Brat DJ, Cooper LAD (2018). Predicting cancer outcomes from histology and genomics using convolutional networks. Proc Natl Acad Sci U S A.

[18] Ching T, Zhu X, Garmire LX. Cox-nnet: An artificial neural network method for prognosis prediction of high-throughput omics data. PLoS Comput Biol. 2018;14(4):e1006076. 10.1371/journal.pcbi.1006076 PMC5909924 29634719

[19] Katzman JL, Shaham U, Cloninger A, Bates J, Jiang TT, Kluger Y. DeepSurv: personalized treatment recommender system using a Cox proportional hazards deep neural network. BMC Med Res Methodol. 2018;18:24. 10.1186/s12874-018-0482-1 PMC5828433 29482517

[20] Liou CY, Cheng WC, Liou JW, Liou DR (2014). Autoencoder for words. Neurocomputing.

[21] Hinton GE, Salakhutdinov RR (2006). Reducing the dimensionality of data with neural networks. Science.

[22] Van Der Maaten L, Postma E, den Herik V (2009). Dimensionality reduction: a comparative. J Mach Learn Res.

[23] Sakurada M, Yairi T (2014). Anomaly detection using autoencoders with nonlinear dimensionality reduction. Proceedings of the MLSDA 2014 2nd Workshop on Machine Learning for Sensory Data Analysis: 2014: ACM.

[24] Wang W, Huang Y, Wang YZ, Wang L (2014). Generalized Autoencoder: A Neural Network Framework for Dimensionality Reduction. 2014 Ieee Conference on Computer Vision and Pattern Recognition Workshops (Cvprw).

[25] Chaudhary K, Poirion OB, Lu L, Garmire LX (2018). Deep learning-based multi-Omics integration robustly predicts survival in liver Cancer. Clin Cancer Res.

[26] Kaufman L, Rousseeuw PJ (1990). Partitioning around medoids (program pam). Finding groups in data: an introduction to cluster analysis.

[27] Efron B (1988). Logistic-regression, survival analysis, and the Kaplan-Meier curve. J Am Stat Assoc.

[28] Alexandrov LB, Nik-Zainal S, Wedge DC, Aparicio SAJR, Behjati S, Biankin AV, Bignell GR, Bolli N, Borg A, Borresen-Dale AL (2013). Signatures of mutational processes in human cancer. Nature.

[29] Yuan J, Hegde PS, Clynes R, Foukas PG, Harari A, Kleen TO, Kvistborg P, Maccalli C, Maecker HT, Page DB, et al. Novel technologies and emerging biomarkers for personalized cancer immunotherapy. J Immunother Cancer. 2016;4:3. 10.1186/s40425-016-0107-3 PMC4717548 26788324

[30] Birkbak NJ, Kochupurakkal B, Izarzugaza JM, Eklund AC, Li Y, Liu J, Szallasi Z, Matulonis UA, Richardson AL, Iglehart JD (2013). Tumor mutation burden forecasts outcome in ovarian cancer with BRCA1 or BRCA2 mutations. PLos one.

[31] Chalmers ZR, Connelly CF, Fabrizio D, Gay L, Ali SM, Ennis R, Schrock A, Campbell B, Shlien A, Chmielecki J (2017). Analysis of 100,000 human cancer genomes reveals the landscape of tumor mutational burden. Genome Med.

[32] Spigel DR, Schrock AB, Fabrizio D, Frampton GM, Sun J, He J, Gowen K, Johnson ML, Bauer TM, Kalemkerian GP (2016). Total mutation burden (TMB) in lung cancer (LC) and relationship with response to PD-1/PD-L1 targeted therapies. American Society of Clinical Oncology.

[33] Goodman AM, Kato S, Bazhenova L, Patel SP, Frampton GM, Miller V, Stephens PJ, Daniels GA, Kurzrock R (2017). Tumor mutational burden as an independent predictor of response to immunotherapy in diverse cancers. Mol Cancer Ther.

[34] Simpson D, Ferguson R, Martinez CN, Kazlow E, Moran U, Heguy A, Hanniford D, Hernando E, Osman I, Kirchhoff T (2017). Mutation burden as a potential prognostic marker of melanoma progression and survival. American Society of Clinical Oncology.

[35] Cox D (1972). Regression models and life tables. Statist Soc B.

[36] Simon N, Friedman J, Hastie T, Tibshirani R (2011). Regularization paths for Cox's proportional hazards model via coordinate descent. J Stat Softw.

[37] Ishwaran H, Kogalur UB, Blackstone EH, Lauer MS (2008). Random survival forests. Ann Appl Stat.

[38] Anderson MJ (2001). A new method for non-parametric multivariate analysis of variance. Austral Ecology.

[39] Hoerl AE, Kennard RW (2000). Ridge regression: biased estimation for nonorthogonal problems. Technometrics.

[40] Tibshirani R (1996). Regression shrinkage and selection via the Lasso. J Royal Stat Soc Series B-Methodological.

[41] Zou H, Hastie T (2005). Regularization and variable selection via the elastic net. J Royal Stat Soc Series B-Statistical Methodology.

[42] Nitanda A (2014). Stochastic proximal gradient descent with acceleration techniques. Advances in Neural Information Processing Systems.

[43] Bottou L (2010). Large-Scale Machine Learning with Stochastic Gradient Descent. Compstat'2010: 19th International Conference on Computational Statistics.

[44] Kingma DP, Ba JL (2014). Adam: A method for stochastic optimization. Proc 3rd Int Conf Learn Representations.

[45] Sobol IM (1976). Uniformly distributed sequences with an additional uniform property. USSR Computational Mathematics Mathematical Physics.

[46] Claesen M, Simm J, Popovic D, Moreau Y, De Moor B (2014). Easy hyperparameter search using Optunity. arXiv preprint.

[47] Pourhoseingholi MA, Baghestani AR, MJG V (2012). How to control confounding effects by statistical analysis. Gastroenterol Hepatol Bed Bench.

[48] Brentnall AR, Cuzick J (2018). Use of the concordance index for predictors of censored survival data. Stat Methods Med Res.

[49] Mayr A, Schmid M (2014). Boosting the Concordance Index for Survival Data - A Unified Framework To Derive and Evaluate Biomarker Combinations. PLoS One.

[50] Gerds TA, Kattan MW, Schumacher M, Yu C (2013). Estimating a time-dependent concordance index for survival prediction models with covariate dependent censoring. Stat Med.

[51] Mann HB, Whitney DR. On a test of whether one of two random variables is stochastically larger than the other. Ann Mathematical Stat. 1947;18(1):50–60.

[52] Wilcoxon F (1945). Individual comparisons by ranking methods. Biom Bull.

[53] Steck H, Krishnapuram B, Dehing-oberije C, Lambin P, Raykar VC (2008). On ranking in survival analysis: bounds on the concordance index. Advances in neural information processing systems.

[54] Mantel N (1966). Evaluation of survival data and two new rank order statistics arising in its consideration. Cancer Chemother Rep.

[55] Peto R, Peto J. Asymptotically efficient rank invariant test procedures. J Royal Stat Soc Series A. 1972;135(2):185–207.

[56] Harrington D. Linear rank tests in survival analysis. Encyclopedia Biostatist. 2005;4:1-13.

[57] Hsu H, Lachenbruch PA (2014). Paired t test. Wiley StatsRef: Statistics Reference Online.

[58] David HA, Gunnink JL (1997). The paired t test under artificial pairing. Am Stat.

[59] Pinheiro J, Bates D, DebRoy S, Sarkar D, Team RC (2007). Linear and nonlinear mixed effects models.

[60] Reese RA, Welsh KB, Galecki AT (2008). Linear mixed models: a practical guide using statistical software. J Royal Stat Soc Series a-Stat Soc.

[61] Fodor IK (2002). JCfASC, Lawrence Livermore National Laboratory: A survey of dimension reduction techniques.

[62] Tan SF, Mavrovouniotis ML (1995). Reducing data dimensionality through optimizing neural-network inputs. AICHE J.

[63] Rousseeuw PJ (1987). Silhouettes - a graphical aid to the interpretation and validation of cluster-analysis. J Comput Appl Math.

[64] Kodinariya TM, Makwana PR (2013). Review on determining number of Cluster in K-Means Clustering. Int J.

[65] Poggio T, Mhaskar H, Rosasco L, Miranda B, Liao Q (2017). Why and when can deep-but not shallow-networks avoid the curse of dimensionality: a review. Int J Autom Comput.

[66] Owada-Ozaki Y, Muto S, Takagi H, Inoue T, Watanabe Y, Fukuhara M, Yamaura T, Okabe N, Matsumura Y, Hasegawa T (2018). Prognostic impact of tumor mutation burden in patients with completely resected non-small cell lung Cancer: brief report. J Thorac Oncol.

[67] Naidoo J, Wang X, Woo KM, Iyriboz T, Halpenny D, Cunningham J, Chaft JE, Segal NH, Callahan MK, Lesokhin AM (2017). Pneumonitis in Patients Treated With Anti-Programmed Death-1/Programmed Death Ligand 1 Therapy. J Clin Oncol.

[68] Huang Z, Han Z, Parwani A, Huang K, Li ZB. Predicting response to neoadjuvant chemotherapy in HER2-positive breast cancer using machine learning models with combined tissue imaging and clinical features. Laboratory investigation. 2019;99.

[69] Huang Z, Tgavalekos K, Zhao C (2020). 221: AI-driven forecasting of mean pulmonary artery pressure for the management of cardiac patients. Crit Care Med.

[70] Wang T, Johnson TS, Shao W, Lu Z, Helm BR, Zhang J, Huang K. BERMUDA: a novel deep transfer learning method for single-cell RNA sequencing batch correction reveals hidden high-resolution cellular subtypes. Genome Biol. 2019;20(1):1-15. 10.1186/s13059-019-1764-6 PMC6691531 31405383

