# Supplementary material for: Deep learning-based cancer survival prognosis from RNA-seq data: approaches and evaluations
Source: BMC Med Genomics. 2020 Apr 3;13(Suppl 5):41. doi: 10.1186/s12920-020-0686-1 (PMC7118823; doi:10.1186/s12920-020-0686-1)
Supplement: Supplementary file 1 — Additional file 1: Figure S1. An example framework of the AECOX model with four hidden layers. Table S1. The network design of AECOX. Table S2. The hyper-parameters of AECOX to be searched. Table S3. Performances of testing set in TCGA Kidney Renal Clear Cell Carcinoma (KIRC) dataset. Bolded texts indicated optimal results among all models. Table S4. Individual model correlations (Pearson ρ) of mean TMB (Fig. 2). Figure S2. Relationship between concordance index and median TMB. Pearson ρ = − 0.30 (p-value = 7.75E-02). Figure S3. Relationship between concordance index and max TMB. Pearson ρ = − 0.40 (p-value = 1.68E-02). Figure S4. Relationship between concordance index and 20% tail TMB. Pearson ρ = − 0.32 (p-value = 5.51E-02). Figure S5. Relationship between concordance index and 10% tail TMB. Pearson ρ = − 0.32 (p-value = 5.93E-02). Figure S6. Relationship between concordance index and 5% tail TMB. Pearson ρ = − 0.30 (p-value = 7.45E-02). Table S5. Fine-tuned hyper-parameters of Cox-nnet (L2 penalty weight λ) across 12 cancer types and 5 experiments (folds). Table S6. Fine-tuned hyper-parameters of DeepSurv across 12 cancer types and 5 experiments (folds). Table S7. Fine-tuned hyper-parameters of AECOX across 12 cancer types and 5 experiments (folds). Note that we fixed λ2 = 0 to only impose L2 sparsity. Table S8. Fine-tuned hyper-parameters of Random Survival Forest (RSF) (number of the trees) across 12 cancer types and 5 experiments (folds). Table S9. Fine-tuned hyper-parameters of SVM (α, weight of penalizing the squared hinge loss in the objective function) across 12 cancer types and 5 experiments (folds). Table S10. Model-wised performances comparison at pan-cancer level (12 TCGA (The Cancer Genome Atlas) cancer types) by pairwise paired t-test, according to metrics concordance index and p-value of log-rank test. Note that for concordance index, larger t-statistic/coefficient indicated better performance at pan-cancer level, while the p-value of log-rank test [file 12920_2020_686_MOESM1_ESM.docx]

**Deep Learning-based Cancer Survival Prognosis from RNA-seq Data: Approaches and Evaluations**

**Supplementary Material**

**1. List of TCGA Cancer Used**

The specific cancers analyzed in this paper were (1) Urothelial Bladder Carcinoma (BLCA); (2) Breast Invasive Carcinoma (BRCA); (3) Cervical Squamous Cell Carcinoma and Endocervical Adenocarcinoma (CESC); (4) Head-Neck Squamous Cell Carcinoma (HNSC); (5) Kidney Renal Clear Cell Carcinoma (KIRC); (6) Kidney Renal Papillary Cell Carcinoma (KIRP); (7) Liver Hepatocellular Carcinoma (LIHC); (8) Lung Adenocarcinoma (LUAD); (9) Lung Squamous Cell Carcinoma (LUSC); (10) Ovarian Cancer (OV); (11) Pancreatic Adenocarcinoma (PAAD); and (12) Stomach Adenocarcinoma (STAD). All mRNA-seq data were based on illuminahiseq_rnaseqv2-RSEM_genes_normalized from Broad GDAC Firehose (<https://gdac.broadinstitute.org/>) transcriptomic data as the inputs to the models.

**2. Online Method AECOX: AutoEncoder with COX Regression Network**

Patient survival prognosis was often performed with small datasets due to the limited collection of clinical data, *i.e.* the number of samples are much less than the number of covariates (features). Thus, training with small amount of patients compared to large amount of features (such as mRNA-seq data) tends to introduce overfitting issues [1]. One way to overcome this issue was to reduce the dimension of inputs, several algorithms such as Principle Component Analysis (PCA) [2], Singular Value Decomposition (SVD) [3, 4], and Non-negative Matrix Factorization (NMF) [5] could be applied. As a new competitive deep learning method, the autoencoder (AE), which is a family of neural networks, can find efficient lower dimensional representations in unsupervised or supervised learning fashions [6]. It also provides a desirable model in different research areas, for example, denoising task [7], generative model as variational autoencoder [8, 9], etc. To this end, we took the advantages of dimensionality reduction in AE, proposed a new algorithm to perform cancer survival prognosis with simultaneous learning for lower dimensional representation of inputs, named AECOX (AutoEncoder with COX regression network). In AECOX (Fig. S1), the code from AE will link to a Cox regression layer for the survival prognosis. Both losses from the AE networks and Cox regression layer will be used to train the entire network weights through back-propagation.


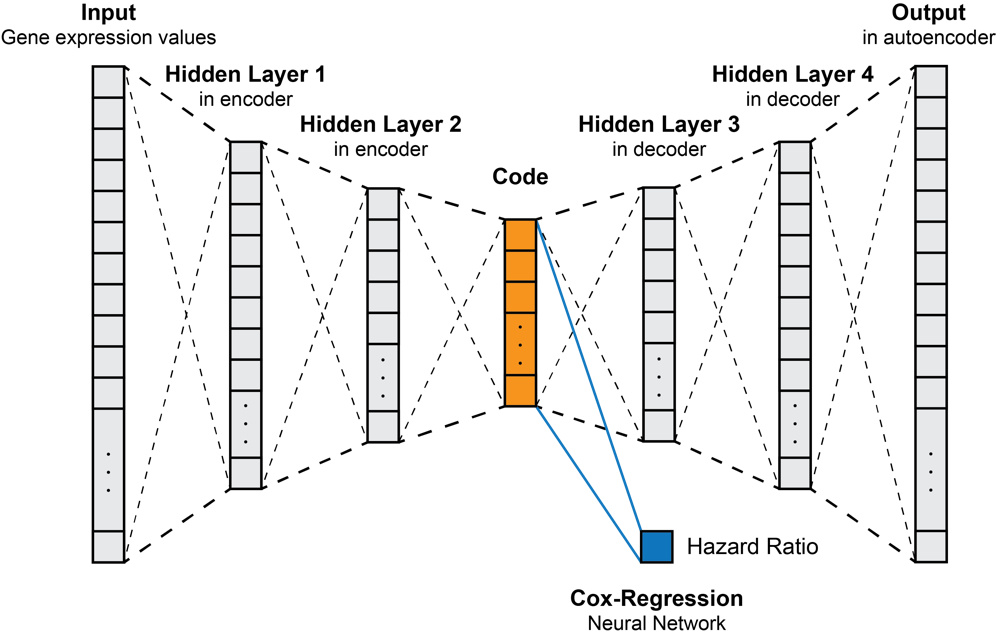


Fig. S1. An example framework of the AECOX model with four hidden layers. Rectangle with boxes are vector representations of input-output layers and hidden layers. Grey and Orange rectangles are autoencoder networks, connected by weights in dashed black lines. Blue box is the regression result of single-layer neural network with Cox regression, also known as the hazards ratio. By treating the code as input (orange), blue lines are representing for single layer perceptron (neural network).

**2.1. AECOX Network Design and Parameter Choosing**

Taking an example of The Cancer Genome Atlas (TCGA) Kidney Renal Clear Cell Carcinoma (KIRC) mRNA-seq data with four hidden layers, Table S1 described the network design of our AECOX model. Input layer is with dimension 17,870 after pre-processing. Two hidden layer with activation function from each side of the autoencoder was adopted in order to introduce non-linearity. We set the dimension of hidden layer 1, 2, 3, 4, and code be 4096, 512, 512, 4096, and 16, respectively. In general, dimensions of each layer are: input layer > hidden layer 1 = hidden layer 4 > hidden layer 2 = hidden layer 3 > code.

We introduced L1 lasso, L2 ridge, elastic net, and dropout regularization as the penalties to the network. Sobol solver [10] was adopted to find optimal hyper-parameters for the loss function. Rectified linear unit (ReLU) [11] was applied as the activation functions after hidden layers of autoencoder part in order to introduce the non-linearity. Hyperbolic tangent (tanh) activation function was used in Cox regression neural network part in order to normalize the outputs within the range $[-1, 1]$.

| **Autoencoder** | | |
| --- | --- | --- |
| Network Layers | Nodes | Activation Function |
| Input Layer | 17,870 |  |
| Hidden Layer 1 | 4,096 | ReLU |
| Hidden Layer 2 | 512 | ReLU |
| Code* | 16 |  |
| Hidden Layer 3 | 512 | ReLU |
| Hidden Layer 4 | 4,096 | ReLU |
| Output Layer | 17,870 |  |
| **Cox Regression Neural Network** | | |
| Network Layers | Nodes | Activation Function |
| Code* | 16 | Tanh |
| Cox Regression Output | 1 |  |

Table S1. The network design of AECOX. Non-linear activation functions are followed right after each linear-transformation layer to introduce the non-linearity. Example input is TCGA Kidney Renal Clear Cell Carcinoma (KIRC) mRNA-seq data with 17,870 features (genes). Objects with * symbol means they share the same memory space.

**2.2. Implementation and Hyper-parameters of AECOX**

AECOX was implemented in PyTorch 0.4.0. The network structure can be with 0, 2, 4, 6, 8 hidden layers (specific number of hidden layers was determined by hyper-parameter searching). Adaptive Moment Estimation (Adam) optimizer [12] was adopted in this work for the learning process. To guarantee the convergence of training processes, we set the number of epochs be 300 for training. The mini-batch technique with size 256 was adopted while training.

As mentioned in the main article, the training, validation, and testing sets were splitted from dataset into 60%, 20%, 20%, respectively. To find the optimal hyper-parameters, training set and validation set were used, while the optimal hyper-parameters were the arguments that maximize the concordance index in validation set. Table S2 shows the hyper-parameters that need to be determined. After the hyper-parameters were determined, model performances were evaluated on the testing set (*e.g.*, Table S3). To find the optimal hyper-parameters, 100 iterations are performed for each dataset, where each iteration represents a 300-epochs training.

| **Hyper-parameters** | | |
| --- | --- | --- |
| Hyper-parameter | Value constraints | Explanation |
| Log learning rate | [-5.5, -3.5] | This learning rate is in ${log}_{10}$ scale. |
| $\lambda_{1}$ | [0, 1] | Adjusting the learning direction by balancing autoencoder loss and Cox regression loss. |
| ${log}_{10}{(\lambda}_{2})$ | [-6, -4] | L1 penalty weight in ${log}_{10}$ scale. |
| ${log}_{10}{(\lambda}_{3})$ | [-6, -4] | L2 penalty weight in ${log}_{10}$ scale. |
| Dropout rate | [0, 0.2] | Dropout rate applied to each hidden layer, if Dropout penalty is selected. |
| Hidden Layers | {0, 2, 4, 6, 8} | Number of hidden layers. If 4 is adopted, then 2 for encoder, 2 for decoder. |
| Penalty | {Lasso, Ridge, Elastic Net, Dropout} | If Lasso, then $\lambda_{3}=0$, Dropout rate $=0$; If Ridge, then $\lambda_{2}=0$, Dropout rate $=0$; If Elastic Net, then Dropout rate $=0$; If Dropout, then $\lambda_{2}=\lambda_{3}=0$. |

Table S2. The hyper-parameters of AECOX to be searched.

In AECOX model's loss function, the regularization weights $\lambda_{2}$ and $\lambda_{3}$ introduced the L1 and L2 norm, while a proper choice of $\lambda_{1}$ guaranteed the back-propagation can simultaneously reduce both the loss of autoencoder and Cox regression effectively. We adopted random hyper-parameter optimization search [13] with Python package Optunity [14], namely, the Sobol solver [10]. In TCGA Kidney Renal Clear Cell Carcinoma (KIRC) dataset, we found learning rate $=5.717\times{10}^{-5}$, $\lambda_{1}=0.6054$, $\lambda_{2}=0$, $\lambda_{3}=1.319\times{10}^{-6}$, number of hidden layers $=0$, and sparsity to be “Ridge” (L2) gave the optimal validation performances. The searching result of hyper-parameters, especially the cost from autoencoder with $\lambda_{1}$ contributions, implied that the decoder could help us to contribute higher performances rather than a direct feed-forward neural network.

**2.3. Objective Function**

As mentioned in the main article, we defined our loss function for AECOX be

${\hat{\Theta}=argmin}_{\Theta}\left\{ \lambda_{1}\mathrm{MSE}\left( X_{input},X_{output} \right)+\left( 1-\lambda_{1} \right)\sum_{i:C_{i}=1} \left( \sum_{k=1}^{K} \beta_{k}X_{ik}-\log\left( \sum_{j:Y_{j}\geq Y_{i}} \theta_{j} \right) \right)+\lambda_{2}\left\| \Theta\right\|_{1}+\lambda_{3}\left\| \Theta\right\|_{2}^{2} \right\}$ (8)

Where $\Theta$ was the network weights of the entire AECOX model. $X_{i}$ was the code trained from autoencoder with respect to $i^{th}$ patient as defined previously. $X_{input}$ and $X_{output}$ were the input and output layer of autoencoder with patients in the current mini-batch. $\mathrm{MSE}\left( \cdot\right)$ stood for mean squared error function. This Cox proportional hazards implementation is based on Cox-nnet [15]. Besides, $\lambda_{1}\in[0, 1]$is to adjust the loss penalty in between autoencoder and cox regression in AECOX; $\lambda_{2}\geq0$ and $\lambda_{3}\geq0$ to adjust the penalty of L1 and L2 regularization. Specific value constraints are referred to Table S2.

**1.4. Performance on TCGA Kidney Renal Clear Cell Carcinoma (KIRC) Dataset**

Table S3 presented the testing set performance comparison between methods in TCGA Kidney Renal Clear Cell Carcinoma (KIRC) Dataset. Values are the averaged results by repeating experiments five times each. From the results, AECOX with ridge regularization achieved the best concordance index among all Deep Learning-based approaches. Thus in the main article, we considered AECOX model was with Ridge regularization in default. For other TCGA cancers, performances were shown in Figure 2 in the main article.

| Performances of Testing Set in TCGA-KIRC Dataset | | |  |
| --- | --- | --- | --- |
| Methods | **Concordance Index** | **P-value of Log-rank Test** | |
| AECOX (dropout) | 0.6898 | 0.0237 | |
| AECOX (elastic) | 0.6866 | 0.0098 | |
| AECOX (lasso) | 0.6913 | 0.0299 | |
| AECOX (ridge) | **0.6982** | 0.0136 | |
| Cox-nnet | 0.6945 | **0.0031** | |
| DeepSurv | 0.6741 | 0.0260 | |

Table S3. Performances of testing set in TCGA Kidney Renal Clear Cell Carcinoma (KIRC) dataset. Bolded texts indicated optimal results among all models.

**3. Relationship between Cancer Prognosis Performances and Tumor Mutation Burden**

TMB values were collected and calculated based on mutation annotation files (MAFs) from Broad GDAC Firehose (http://gdac.broadinstitute.org/), according to the number of HUGO (Human Genome Organization) gene symbols’ existence for every single available patient. For each cancer, since not all patients had their MAF collected, patients with TMB values were the subset of the patients we used for survival prognosis. In our experiments, only patients with primary cancer were considered. The individual model correlations (Pearson $\rho$) are range from $-0.454$ to $-0.446$, as shown in Table S4.

|  | **Cox-nnet** | **DeepSurv** | **AECOX** |
| --- | --- | --- | --- |
| Pearson $\rho$ | -0.4457 | -0.4538 | -0.4466 |

Table S4. Individual model correlations (Pearson $\rho$) of mean TMB (Fig. 2).

Instead of mean TMB, other TMB statistics were shown as below:


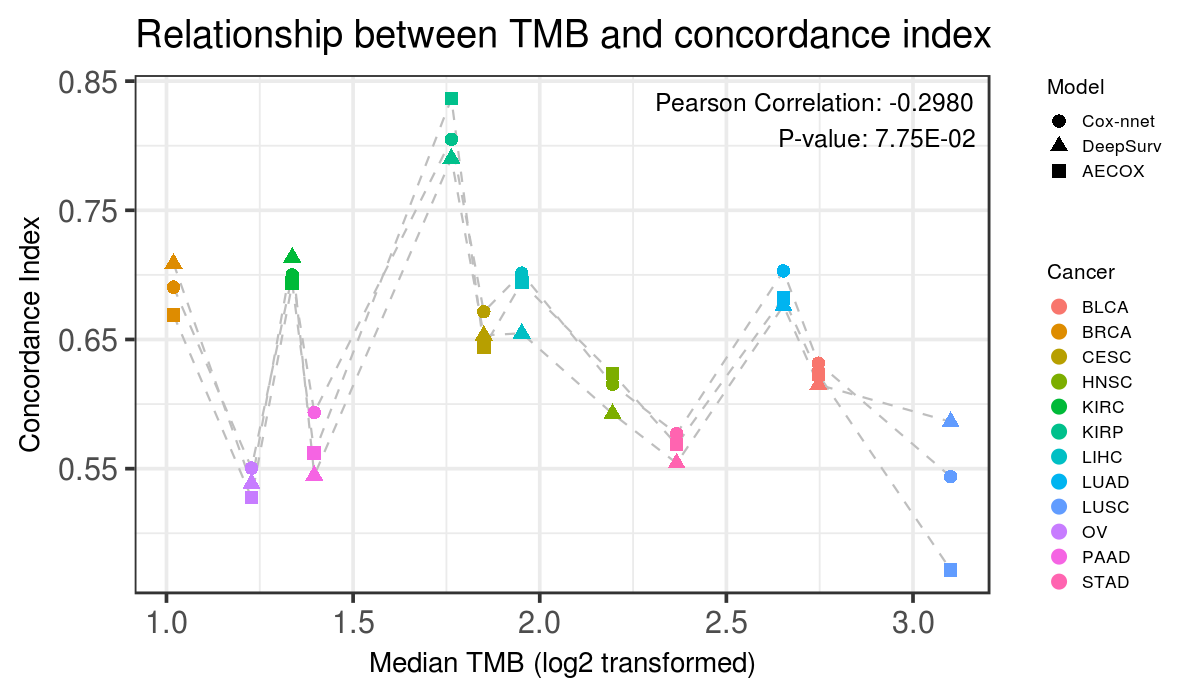


Fig. S2. Relationship between concordance index and median TMB. Pearson $\rho=-0.30$ (p-value = 7.75E-02).


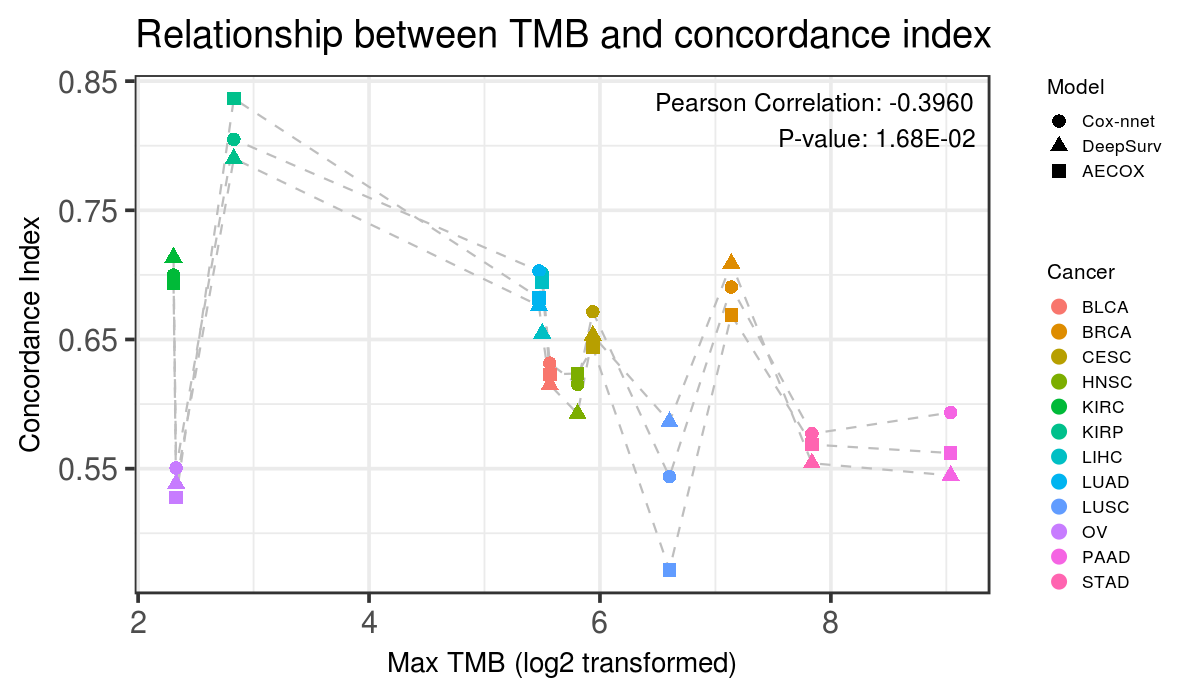


Fig. S3. Relationship between concordance index and max TMB. Pearson $\rho=-0.40$ (p-value = 1.68E-02).


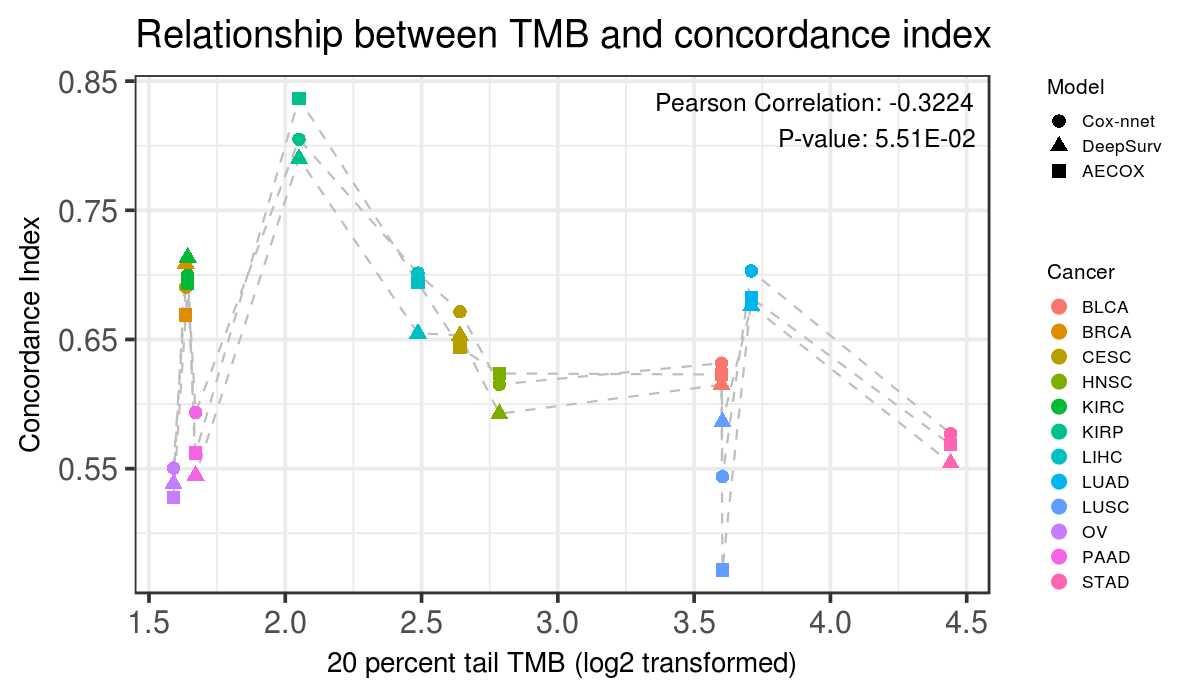


Fig. S4. Relationship between concordance index and 20% tail TMB. Pearson $\rho=-0.32$ (p-value = 5.51E-02).


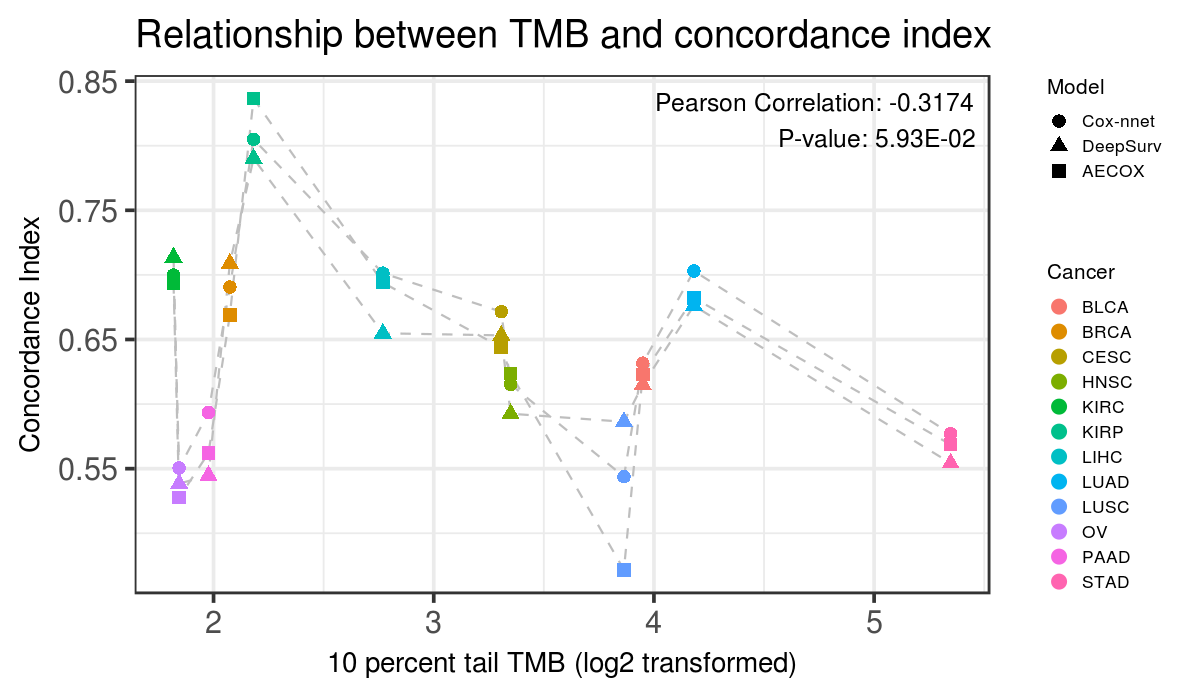


Fig. S5. Relationship between concordance index and 10% tail TMB. Pearson $\rho=-0.32$ (p-value = 5.93E-02).


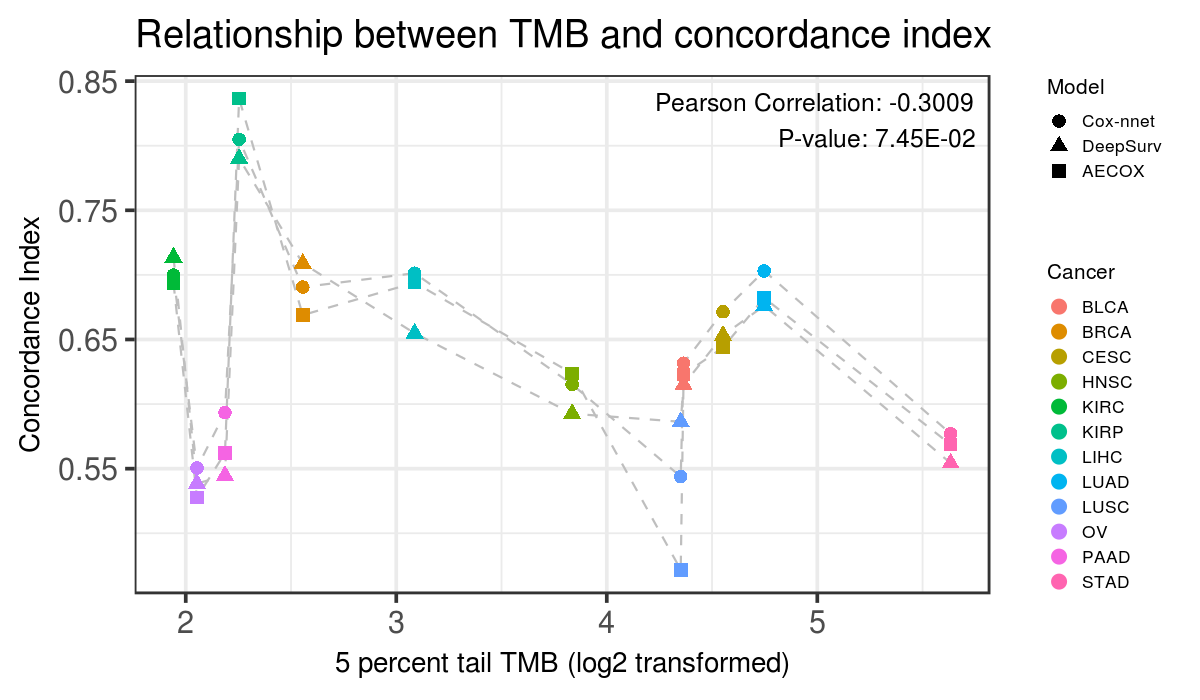


Fig. S6. Relationship between concordance index and 5% tail TMB. Pearson $\rho=-0.30$ (p-value = 7.45E-02).

All aforementioned figures showed that concordance indices are negatively correlated with all TMB statistics.

**4. Associated Fine-tuned Hyper-parameters**

**4.1. Fine-tuned hyper-parameters of Cox-nnet (L2 penalty weight** $\boldsymbol{\lambda}$**)**

| Cancer | fold 1 | fold 2 | fold 3 | fold 4 | fold 5 |
| --- | --- | --- | --- | --- | --- |
| BLCA | 0.130029 | 0.00247875 | 0.00247875 | 0.251579 | 0.251579 |
| BRCA | 0.0347353 | 0.0347353 | 0.0347353 | 0.00247875 | 0.0672055 |
| CESC | 0.251579 | 0.251579 | 0.251579 | 0.251579 | 0.00479587 |
| HNSC | 0.0347353 | 0.486752 | 0.130029 | 0.00927901 | 0.00479587 |
| KIRC | 0.0672055 | 0.486752 | 0.130029 | 0.941765 | 0.130029 |
| KIRP | 0.0672055 | 0.00247875 | 0.130029 | 0.251579 | 0.00479587 |
| LIHC | 0.251579 | 0.251579 | 0.130029 | 0.251579 | 0.251579 |
| LUAD | 0.251579 | 0.251579 | 0.486752 | 0.0672055 | 0.00247875 |
| LUSC | 0.486752 | 0.486752 | 0.941765 | 0.486752 | 0.486752 |
| OV | 0.251579 | 0.0672055 | 0.017953 | 0.130029 | 0.251579 |
| PAAD | 0.486752 | 0.00247875 | 0.00479587 | 0.251579 | 0.941765 |
| STAD | 0.486752 | 0.017953 | 0.486752 | 0.00479587 | 0.00927901 |

Table S5. Fine-tuned hyper-parameters of Cox-nnet (L2 penalty weight $\lambda$) across 12 cancer types and 5 experiments (folds).

**4.2. Fine-tuned hyper-parameters of DeepSurv**

| Cancer | Learning rate | Learning rate decay | Momentum | L2 penalty | Dropout rate | Number of hidden layers | Hidden layer size |
| --- | --- | --- | --- | --- | --- | --- | --- |
| BLCA (fold 1) | 2.87E-03 | 6.60E-04 | 8.49E-01 | 7.57E-04 | 9.63E-02 | 1 | 42 |
| BLCA (fold 2) | 4.88E-04 | 1.80E-04 | 8.09E-01 | 2.55E-04 | 5.91E-02 | 3 | 43 |
| BLCA (fold 3) | 6.21E-04 | 5.24E-04 | 9.45E-01 | 3.21E-04 | 1.42E-01 | 3 | 45 |
| BLCA (fold 4) | 1.06E-04 | 1.92E-04 | 8.70E-01 | 5.30E-04 | 4.55E-02 | 2 | 38 |
| BLCA (fold 5) | 4.16E-04 | 1.57E-04 | 9.12E-01 | 1.70E-04 | 1.56E-01 | 4 | 37 |
| BRCA (fold 1) | 6.36E-03 | 3.44E-04 | 9.19E-01 | 7.82E-04 | 1.72E-02 | 2 | 38 |
| BRCA (fold 2) | 1.99E-04 | 9.36E-04 | 8.44E-01 | 2.92E-05 | 2.14E-02 | 4 | 46 |
| BRCA (fold 3) | 5.26E-03 | 9.57E-04 | 8.24E-01 | 6.31E-04 | 4.94E-02 | 3 | 36 |
| BRCA (fold 4) | 4.39E-03 | 1.04E-04 | 9.14E-01 | 9.43E-04 | 6.53E-02 | 2 | 45 |
| BRCA (fold 5) | 6.46E-04 | 6.73E-04 | 9.26E-01 | 6.02E-04 | 1.99E-01 | 3 | 31 |
| CESC (fold 1) | 4.65E-05 | 4.58E-04 | 8.50E-01 | 6.58E-04 | 1.27E-02 | 3 | 32 |
| CESC (fold 2) | 9.70E-05 | 2.82E-04 | 9.32E-01 | 7.91E-04 | 4.68E-01 | 2 | 32 |
| CESC (fold 3) | 6.24E-05 | 7.95E-04 | 8.81E-01 | 4.84E-04 | 4.25E-01 | 1 | 36 |
| CESC (fold 4) | 4.91E-04 | 4.95E-05 | 9.36E-01 | 1.93E-04 | 3.32E-01 | 4 | 48 |
| CESC (fold 5) | 4.65E-05 | 4.58E-04 | 8.50E-01 | 6.58E-04 | 1.27E-02 | 3 | 32 |
| HNSC (fold 1) | 3.05E-05 | 1.85E-05 | 8.75E-01 | 7.80E-04 | 3.79E-01 | 2 | 43 |
| HNSC (fold 2) | 3.14E-04 | 2.05E-04 | 9.44E-01 | 3.11E-04 | 4.14E-01 | 1 | 45 |
| HNSC (fold 3) | 1.88E-04 | 4.36E-04 | 8.08E-01 | 8.89E-04 | 1.23E-01 | 4 | 42 |
| HNSC (fold 4) | 7.62E-05 | 7.35E-04 | 8.24E-01 | 7.87E-04 | 4.16E-01 | 4 | 42 |
| HNSC (fold 5) | 1.37E-04 | 3.11E-05 | 8.04E-01 | 5.63E-04 | 2.96E-01 | 4 | 32 |
| KIRC (fold 1) | 4.44E-05 | 6.45E-04 | 9.48E-01 | 3.09E-04 | 8.85E-02 | 4 | 44 |
| KIRC (fold 2) | 9.07E-05 | 9.88E-04 | 8.38E-01 | 3.52E-04 | 2.20E-01 | 1 | 31 |
| KIRC (fold 3) | 3.27E-04 | 9.26E-04 | 9.03E-01 | 4.27E-05 | 3.74E-01 | 2 | 44 |
| KIRC (fold 4) | 3.78E-05 | 4.21E-04 | 9.17E-01 | 5.64E-04 | 1.47E-01 | 3 | 46 |
| KIRC (fold 5) | 2.69E-05 | 2.21E-04 | 8.30E-01 | 2.34E-04 | 3.13E-01 | 2 | 44 |
| KIRP (fold 1) | 3.12E-04 | 8.04E-04 | 8.45E-01 | 6.22E-04 | 1.14E-01 | 3 | 30 |
| KIRP (fold 2) | 7.37E-05 | 6.88E-04 | 9.39E-01 | 4.44E-04 | 1.56E-01 | 3 | 34 |
| KIRP (fold 3) | 2.74E-05 | 5.91E-04 | 8.31E-01 | 4.25E-04 | 2.63E-01 | 4 | 48 |
| KIRP (fold 4) | 5.53E-05 | 3.79E-04 | 8.35E-01 | 8.34E-04 | 1.14E-01 | 1 | 49 |
| KIRP (fold 5) | 1.54E-05 | 8.12E-04 | 9.00E-01 | 8.95E-04 | 8.32E-02 | 2 | 43 |
| LIHC (fold 1) | 2.97E-05 | 4.01E-04 | 8.40E-01 | 3.40E-05 | 4.69E-01 | 1 | 44 |
| LIHC (fold 2) | 1.02E-03 | 5.61E-04 | 9.18E-01 | 9.46E-04 | 4.22E-01 | 3 | 44 |
| LIHC (fold 3) | 1.08E-04 | 9.17E-04 | 8.28E-01 | 1.57E-04 | 1.51E-01 | 3 | 48 |
| LIHC (fold 4) | 1.94E-05 | 8.35E-04 | 9.23E-01 | 2.20E-04 | 4.39E-01 | 2 | 48 |
| LIHC (fold 5) | 3.16E-05 | 4.29E-04 | 9.03E-01 | 2.57E-04 | 2.55E-01 | 3 | 41 |
| LUAD (fold 1) | 2.23E-05 | 8.82E-04 | 8.57E-01 | 7.64E-04 | 2.57E-01 | 2 | 48 |
| LUAD (fold 2) | 4.97E-05 | 7.97E-04 | 9.44E-01 | 2.30E-04 | 2.61E-01 | 4 | 33 |
| LUAD (fold 3) | 5.28E-05 | 7.13E-04 | 8.99E-01 | 7.98E-04 | 4.84E-01 | 3 | 48 |
| LUAD (fold 4) | 4.63E-04 | 5.44E-04 | 8.68E-01 | 7.74E-04 | 4.73E-01 | 1 | 43 |
| LUAD (fold 5) | 5.32E-05 | 2.92E-04 | 8.13E-01 | 1.20E-04 | 4.17E-02 | 1 | 33 |
| LUSC (fold 1) | 4.35E-05 | 6.61E-04 | 8.31E-01 | 9.53E-04 | 3.56E-01 | 3 | 32 |
| LUSC (fold 2) | 9.02E-04 | 7.48E-04 | 8.23E-01 | 9.63E-04 | 3.86E-01 | 4 | 39 |
| LUSC (fold 3) | 5.30E-03 | 8.11E-04 | 9.20E-01 | 8.15E-04 | 3.42E-01 | 4 | 31 |
| LUSC (fold 4) | 1.52E-05 | 1.81E-04 | 8.14E-01 | 2.16E-04 | 3.82E-01 | 4 | 38 |
| LUSC (fold 5) | 1.04E-04 | 5.21E-04 | 8.18E-01 | 6.80E-04 | 2.67E-02 | 2 | 46 |
| OV (fold 1) | 5.39E-05 | 5.69E-04 | 8.45E-01 | 7.37E-04 | 1.55E-01 | 4 | 43 |
| OV (fold 2) | 2.84E-05 | 9.71E-04 | 8.03E-01 | 8.92E-04 | 3.87E-01 | 1 | 46 |
| OV (fold 3) | 8.61E-04 | 5.17E-04 | 8.34E-01 | 7.07E-04 | 3.34E-01 | 1 | 41 |
| OV (fold 4) | 3.00E-03 | 2.57E-04 | 8.76E-01 | 4.26E-04 | 4.38E-01 | 1 | 44 |
| OV (fold 5) | 1.04E-03 | 1.89E-04 | 9.19E-01 | 6.41E-04 | 4.95E-01 | 1 | 47 |
| PAAD (fold 1) | 3.04E-03 | 6.03E-04 | 9.27E-01 | 7.95E-04 | 3.66E-01 | 4 | 34 |
| PAAD (fold 2) | 1.80E-05 | 8.51E-04 | 8.15E-01 | 5.47E-04 | 2.42E-01 | 1 | 39 |
| PAAD (fold 3) | 1.51E-05 | 4.85E-05 | 9.42E-01 | 2.79E-04 | 2.25E-01 | 3 | 33 |
| PAAD (fold 4) | 8.18E-03 | 4.91E-04 | 8.38E-01 | 7.17E-05 | 1.01E-01 | 4 | 33 |
| PAAD (fold 5) | 4.67E-05 | 1.89E-04 | 8.68E-01 | 7.51E-04 | 1.26E-01 | 4 | 44 |
| STAD (fold 1) | 1.99E-05 | 8.08E-04 | 9.24E-01 | 8.75E-04 | 1.87E-01 | 1 | 31 |
| STAD (fold 2) | 5.93E-03 | 5.07E-04 | 9.10E-01 | 9.22E-04 | 4.95E-01 | 3 | 32 |
| STAD (fold 3) | 3.47E-05 | 1.07E-04 | 8.22E-01 | 5.82E-05 | 1.96E-01 | 3 | 47 |
| STAD (fold 4) | 3.56E-05 | 9.15E-04 | 8.49E-01 | 4.02E-04 | 3.97E-02 | 2 | 41 |
| STAD (fold 5) | 1.33E-05 | 3.39E-04 | 8.43E-01 | 7.54E-04 | 3.59E-01 | 3 | 44 |

Table S6. Fine-tuned hyper-parameters of DeepSurv across 12 cancer types and 5 experiments (folds).

**4.3. Fine-tuned hyper-parameters of AECOX**

| Cancer | Learning rate | Number of hidden layers | $\lambda_{1}$ | $\lambda_{2}$ | $\lambda_{3}$ |
| --- | --- | --- | --- | --- | --- |
| BLCA (fold 1) | 9.17E-06 | 4 | 0.33951172 | 0.00E+00 | 2.47E-06 |
| BLCA (fold 2) | 2.02E-05 | 1 | 0.55704102 | 0.00E+00 | 3.65E-05 |
| BLCA (fold 3) | 3.83E-05 | 4 | 0.20125977 | 0.00E+00 | 9.22E-05 |
| BLCA (fold 4) | 5.37E-05 | 2 | 0.7465332 | 0.00E+00 | 7.58E-05 |
| BLCA (fold 5) | 3.20E-05 | 3 | 0.85868164 | 0.00E+00 | 1.99E-05 |
| BRCA (fold 1) | 0.00018602 | 1 | 0.01080078 | 0.00E+00 | 1.21E-05 |
| BRCA (fold 2) | 0.00021739 | 1 | 0.0369043 | 0.00E+00 | 3.69E-05 |
| BRCA (fold 3) | 3.83E-05 | 4 | 0.20125977 | 0.00E+00 | 9.22E-05 |
| BRCA (fold 4) | 0.00021836 | 0 | 0.33177734 | 0.00E+00 | 7.28E-05 |
| BRCA (fold 5) | 0.00011298 | 1 | 0.08814453 | 0.00E+00 | 3.51E-05 |
| CESC (fold 1) | 7.68E-06 | 4 | 0.71849609 | 0.00E+00 | 6.94E-06 |
| CESC (fold 2) | 4.02E-06 | 2 | 0.1606543 | 0.00E+00 | 6.67E-06 |
| CESC (fold 3) | 1.12E-05 | 3 | 0.6537207 | 0.00E+00 | 4.94E-05 |
| CESC (fold 4) | 3.51E-06 | 1 | 0.11521484 | 0.00E+00 | 8.60E-06 |
| CESC (fold 5) | 0.00013028 | 4 | 0.49033203 | 0.00E+00 | 9.51E-05 |
| HNSC (fold 1) | 5.25E-05 | 3 | 0.26990234 | 0.00E+00 | 1.75E-05 |
| HNSC (fold 2) | 0.00023658 | 4 | 0.86738281 | 0.00E+00 | 5.97E-06 |
| HNSC (fold 3) | 9.84E-05 | 2 | 0.84708008 | 0.00E+00 | 8.04E-06 |
| HNSC (fold 4) | 2.27E-05 | 1 | 0.60634766 | 0.00E+00 | 9.18E-05 |
| HNSC (fold 5) | 0.00025068 | 0 | 0.8103418 | 0.00E+00 | 1.81E-05 |
| KIRC (fold 1) | 6.51E-06 | 4 | 0.412021 | 0.00E+00 | 3.21E-06 |
| KIRC (fold 2) | 6.28E-05 | 3 | 0.97373 | 0.00E+00 | 8.29E-06 |
| KIRC (fold 3) | 8.89E-06 | 2 | 0.394619 | 0.00E+00 | 2.30E-05 |
| KIRC (fold 4) | 5.72E-05 | 0 | 0.60538086 | 0.00E+00 | 1.32E-06 |
| KIRC (fold 5) | 1.34E-05 | 2 | 0.290205 | 0.00E+00 | 1.07E-05 |
| KIRP (fold 1) | 0.00027039 | 0 | 0.06880859 | 0.00E+00 | 1.45E-06 |
| KIRP (fold 2) | 2.39E-05 | 1 | 0.64018555 | 0.00E+00 | 1.27E-05 |
| KIRP (fold 3) | 8.54E-06 | 3 | 0.78810547 | 0.00E+00 | 1.95E-05 |
| KIRP (fold 4) | 7.11E-05 | 0 | 0.35884766 | 0.00E+00 | 2.94E-05 |
| KIRP (fold 5) | 4.25E-06 | 4 | 0.87608398 | 0.00E+00 | 1.54E-05 |
| LIHC (fold 1) | 4.28E-06 | 1 | 0.04270508 | 0.00E+00 | 3.42E-06 |
| LIHC (fold 2) | 4.02E-06 | 2 | 0.1606543 | 0.00E+00 | 6.67E-06 |
| LIHC (fold 3) | 1.96E-05 | 2 | 0.68272461 | 0.00E+00 | 5.75E-05 |
| LIHC (fold 4) | 5.47E-06 | 3 | 0.54447266 | 0.00E+00 | 1.25E-05 |
| LIHC (fold 5) | 0.00017872 | 4 | 0.85288086 | 0.00E+00 | 4.03E-05 |
| LUAD (fold 1) | 1.04E-05 | 2 | 0.32887695 | 0.00E+00 | 8.33E-06 |
| LUAD (fold 2) | 0.00018356 | 0 | 0.97469727 | 0.00E+00 | 6.02E-05 |
| LUAD (fold 3) | 1.80E-05 | 3 | 0.08331055 | 0.00E+00 | 5.26E-05 |
| LUAD (fold 4) | 9.12E-05 | 2 | 0.59861328 | 0.00E+00 | 1.89E-06 |
| LUAD (fold 5) | 9.50E-06 | 2 | 0.14615234 | 0.00E+00 | 9.69E-05 |
| LUSC (fold 1) | 1.10E-05 | 2 | 0.36271484 | 0.00E+00 | 2.69E-05 |
| LUSC (fold 2) | 1.26E-05 | 0 | 0.20802734 | 0.00E+00 | 1.32E-05 |
| LUSC (fold 3) | 5.77E-06 | 0 | 0.03787109 | 0.00E+00 | 2.95E-06 |
| LUSC (fold 4) | 0.0001029 | 0 | 0.94182617 | 0.00E+00 | 5.45E-05 |
| LUSC (fold 5) | 5.19E-06 | 1 | 0.71076172 | 0.00E+00 | 4.27E-05 |
| OV (fold 1) | 2.04E-05 | 2 | 0.45939453 | 0.00E+00 | 1.99E-05 |
| OV (fold 2) | 7.92E-06 | 1 | 0.38495117 | 0.00E+00 | 2.37E-06 |
| OV (fold 3) | 3.20E-05 | 3 | 0.85868164 | 0.00E+00 | 1.99E-05 |
| OV (fold 4) | 1.91E-05 | 2 | 0.21286133 | 0.00E+00 | 8.44E-05 |
| OV (fold 5) | 6.17E-05 | 2 | 0.20416016 | 0.00E+00 | 1.08E-05 |
| PAAD (fold 1) | 1.25E-05 | 1 | 0.73879883 | 0.00E+00 | 2.40E-06 |
| PAAD (fold 2) | 0.0001509 | 2 | 0.07364258 | 0.00E+00 | 6.97E-06 |
| PAAD (fold 3) | 1.17E-05 | 2 | 0.60924805 | 0.00E+00 | 1.12E-06 |
| PAAD (fold 4) | 0.00017635 | 1 | 0.78037109 | 0.00E+00 | 9.23E-06 |
| PAAD (fold 5) | 3.26E-06 | 1 | 0.81952637 | 0.00E+00 | 2.03E-05 |
| STAD (fold 1) | 1.16E-05 | 0 | 0.13551758 | 0.00E+00 | 2.90E-05 |
| STAD (fold 2) | 4.89E-05 | 2 | 0.68755859 | 0.00E+00 | 2.56E-06 |
| STAD (fold 3) | 5.72E-05 | 0 | 0.60538086 | 0.00E+00 | 1.32E-06 |
| STAD (fold 4) | 9.25E-05 | 1 | 0.9650293 | 0.00E+00 | 5.02E-06 |
| STAD (fold 5) | 6.75E-06 | 0 | 0.45069336 | 0.00E+00 | 2.63E-05 |

Table S7. Fine-tuned hyper-parameters of AECOX across 12 cancer types and 5 experiments (folds). Note that we fixed $\lambda_{2}=0$ to only impose L2 sparsity.

**4.4. Fine-tuned hyper-parameters of Random Survival Forest (RSF)**

| Cancer | fold 1 | fold 2 | fold 3 | fold 4 | fold 5 |
| --- | --- | --- | --- | --- | --- |
| BLCA | 32 | 128 | 256 | 32 | 64 |
| BRCA | 64 | 512 | 256 | 32 | 64 |
| CESC | 32 | 16 | 32 | 64 | 4096 |
| HNSC | 32 | 64 | 16 | 128 | 64 |
| KIRC | 32 | 64 | 64 | 128 | 256 |
| KIRP | 16 | 64 | 32 | 128 | 2048 |
| LIHC | 64 | 1024 | 128 | 64 | 64 |
| LUAD | 128 | 32 | 32 | 512 | 1024 |
| LUSC | 64 | 4096 | 32 | 16 | 16 |
| OV | 16 | 16 | 256 | 128 | 1024 |
| PAAD | 64 | 32 | 16 | 64 | 32 |
| STAD | 16 | 256 | 16 | 64 | 16 |

Table S8. Fine-tuned hyper-parameters of Random Survival Forest (RSF) (number of the trees) across 12 cancer types and 5 experiments (folds).

**4.5. Fine-tuned hyper-parameters of SVM**

| Cancer | fold 1 | fold 2 | fold 3 | fold 4 | fold 5 |
| --- | --- | --- | --- | --- | --- |
| BLCA | 0.01 | 0.001 | 0.5 | 0.01 | 0.001 |
| BRCA | 0.001 | 1.00E-05 | 1.00E-08 | 1 | 0.01 |
| CESC | 10 | 0.01 | 0.0001 | 0.001 | 0.001 |
| HNSC | 0.01 | 1.00E-08 | 1.00E-06 | 0.001 | 1.00E-06 |
| KIRC | 1.00E-05 | 0.0001 | 0.0001 | 1.00E-05 | 0.001 |
| KIRP | 1.00E-08 | 0.01 | 1.00E-08 | 1.00E-08 | 0.001 |
| LIHC | 0.0001 | 0.0001 | 0.001 | 100000 | 100 |
| LUAD | 0.001 | 0.0001 | 0.0001 | 0.001 | 1.00E-05 |
| LUSC | 1.00E-05 | 1.00E-08 | 1.00E-08 | 1 | 1.00E-05 |
| OV | 0.01 | 1 | 0.1 | 1.00E-06 | 1.00E-05 |
| PAAD | 0.01 | 0.01 | 10000 | 0.0001 | 0.01 |
| STAD | 1.00E-08 | 500 | 0.001 | 0.0001 | 0.01 |

Table S9. Fine-tuned hyper-parameters of SVM ($\alpha$, weight of penalizing the squared hinge loss in the objective function) across 12 cancer types and 5 experiments (folds).

**5. Model-wised Performances Comparison at Pan-cancer Level (All Models)**

| Distribution 2 | SVM | P | 1.42E-07 | 2.01E-01 | 2.32E-03 | 8.00E-01 | 2.64E-04 | 7.63E-01 | 9.77E-03 | 6.00E-01 | 1.57E-01 | 4.28E-02 | Notes: t denotes the pairwise paired Student’s t-test statistic, P denotes the p-value obtained. | Table S10. Model-wised performances comparison at pan-cancer level (12 TCGA (The Cancer Genome Atlas) cancer types) by pairwise paired t-test, according to metrics concordance index and p-value of log-rank test. Note that for concordance index, larger t-statistic/coefficient indicated better performance at pan-cancer level, while the p-value of log-rank test was on the contrary. |
| --- | --- | --- | --- | --- | --- | --- | --- | --- | --- | --- | --- | --- | --- | --- |
|  |  | t | 5.9747 | -1.2928 | 3.1846 | 0.2550 | 3.8819 | -0.3032 | 2.6705 | -0.5273 | -1.4327 | 2.0699 |  |  |
|  | RSF | P | 9.14E-09 | 5.48E-03 | 9.11E-05 | 6.29E-02 | 2.92E-05 | 3.28E-02 | 5.30E-04 | 1.84E-02 | - | - |  |  |
|  |  | t | 6.6871 | -2.8837 | 4.2011 | -1.8955 | 4.5303 | -2.1861 | 3.6661 | -2.4243 | - | - |  |  |
|  | Cox-PH | P | 1.55E-03 | 6.29E-01 | 4.79E-01 | 5.35E-01 | 1.67E-01 | 8.24E-01 | - | - | 5.30E-04 | 1.84E-02 |  |  |
|  |  | t | 3.3191 | -0.4853 | 0.7132 | 0.6243 | 1.3998 | 0.2232 | - | - | -3.6661 | 2.4243 |  |  |
|  | AECOX | P | 2.04E-03 | 3.74E-01 | 5.03E-01 | 6.07E-01 | - | - | 1.67E-01 | 8.24E-01 | 2.92E-05 | 3.28E-02 |  |  |
|  |  | t | 3.2281 | -0.8962 | -0.6732 | 0.5164 | - | - | -1.3998 | -0.2232 | -4.5303 | 2.1861 |  |  |
|  | DeepSurv | P | 2.32E-03 | 1.67E-01 | - | - | 5.03E-01 | 6.07E-01 | 4.79E-01 | 5.35E-01 | 9.11E-05 | 6.29E-02 |  |  |
|  |  | t | 3.1843 | -1.4006 | - | - | 0.6732 | -0.5164 | -0.7132 | -0.6243 | -4.2011 | 1.8955 |  |  |
|  | | | concordance index | p-value of log-rank test | concordance index | p-value of log-rank test | concordance index | p-value of log-rank test | concordance index | p-value of log-rank test | concordance index | p-value of log-rank test |  |  |
|  |  |  | Cox-nnet | | DeepSurv | | AECOX | | Cox-PH | | RSF | |  |  |
|  |  |  | Distribution 1 | | | | | | | | | |  |  |

| **Distribution 2** | **SVM** | P | 3.74E-06 | 3.29E-01 | 8.43E-03 | 8.45E-01 | 2.63E-03 | 8.04E-01 | 3.07E-02 | 6.49E-01 | 1.70E-01 | 3.63E-02 | Notes: $\beta$ denotes the coefficient (slope) of linear mixed-effects models, P denotes the p-value obtained. | Table S11. Model-wised performances comparison at pan-cancer level (12 TCGA (The Cancer Genome Atlas) cancer types) by linear mixed-effects models test, according to metrics concordance index and p-value of log-rank test. Note that for concordance index, larger t-statistic/coefficient indicated better performance at pan-cancer level, while the p-value of log-rank test was on the contrary. |
| --- | --- | --- | --- | --- | --- | --- | --- | --- | --- | --- | --- | --- | --- | --- |
|  |  | $\beta$ | 0.0474 | -0.0403 | 0.0279 | 0.0086 | 0.0331 | -0.0109 | 0.0216 | -0.0197 | -0.0154 | 0.1026 |  |  |
|  | **RSF** | P | 4.16E-09 | 3.95E-03 | 6.81E-05 | 6.24E-02 | 2.18E-05 | 2.44E-02 | 7.22E-04 | 1.56E-02 | - | - |  |  |
|  |  | $\beta$ | 0.0628 | -0.1430 | 0.0433 | -0.0940 | 0.0485 | -0.1136 | 0.0370 | -0.1223 | - | - |  |  |
|  | **Cox-PH** | P | 3.63E-03 | 6.27E-01 | 5.10E-01 | 5.32E-01 | 2.39E-01 | 8.44E-01 | - | - | 7.22E-04 | 1.56E-02 |  |  |
|  |  | $\beta$ | 0.0257 | -0.0206 | 0.0063 | 0.0283 | 0.0115 | 0.0087 | - | - | -0.0370 | 0.1223 |  |  |
|  | **AECOX** | P | 1.12E-01 | 4.85E-01 | 5.85E-01 | 6.62E-01 | - | - | 2.39E-01 | 8.44E-01 | 2.18E-05 | 2.44E-02 |  |  |
|  |  | $\beta$ | 0.0142 | -0.0294 | -0.0052 | 0.0195 | - | - | -0.0115 | -0.0087 | -0.0485 | 0.1136 |  |  |
|  | **DeepSurv** | P | 1.97E-02 | 2.52E-01 | - | - | 5.85E-01 | 6.62E-01 | 5.10E-01 | 5.32E-01 | 6.81E-05 | 6.24E-02 |  |  |
|  |  | $\beta$ | 0.0195 | -0.0489 | - | - | 0.0052 | -0.0195 | -0.0063 | -0.0283 | -0.0433 | 0.0940 |  |  |
|  | | | concordance index | p-value of log-rank test | concordance index | p-value of log-rank test | concordance index | p-value of log-rank test | concordance index | p-value of log-rank test | concordance index | p-value of log-rank test |  |  |
|  |  |  | **Cox-nnet** | | **DeepSurv** | | **AECOX** | | **Cox-PH** | | **RSF** | |  |  |
|  |  |  | **Distribution 1** | | | | | | | | | |  |  |

**References**

1. Hawkins DM: **The problem of overfitting**. *J Chem Inf Comp Sci* 2004, **44**(1):1-12.

2. Pearson K: **LIII. On lines and planes of closest fit to systems of points in space**. *The London, Edinburgh, and Dublin Philosophical Magazine and Journal of Science* 1901, **2**(11):559-572.

3. Alter O, Brown PO, Botstein D: **Singular value decomposition for genome-wide expression data processing and modeling**. *P Natl Acad Sci USA* 2000, **97**(18):10101-10106.

4. Wall ME, Rechtsteiner A, Rocha LM: **Singular value decomposition and principal component analysis**. In: *A practical approach to microarray data analysis.* Springer; 2003: 91-109.

5. Lee DD, Seung HS: **Algorithms for non-negative matrix factorization**. *Adv Neur In* 2001, **13**:556-562.

6. Liou CY, Cheng WC, Liou JW, Liou DR: **Autoencoder for words**. *Neurocomputing* 2014, **139**:84-96.

7. Tan J, Hammond JH, Hogan DA, Greene CS: **ADAGE-Based Integration of Publicly Available Pseudomonas aeruginosa Gene Expression Data with Denoising Autoencoders Illuminates Microbe-Host Interactions**. *Msystems* 2016, **1**(1).

8. Kingma DP, Welling M: **Auto-encoding variational bayes**. *arXiv preprint arXiv:13126114* 2013.

9. Rezende DJ, Mohamed S, Wierstra D: **Stochastic backpropagation and approximate inference in deep generative models**. *arXiv preprint arXiv:14014082* 2014.

10. Sobol IM: **Uniformly distributed sequences with an additional uniform property**. *USSR Computational Mathematics and Mathematical Physics* 1976, **16**(5):236-242.

11. Nair V, Hinton GE: **Rectified linear units improve restricted boltzmann machines**. In: *Proceedings of the 27th international conference on machine learning (ICML-10): 2010*; 2010: 807-814.

12. Kingma DP, Ba J: **Adam: A method for stochastic optimization**. *Proceedings of the 3rd International Conference on Learning Representations (ICLR)* 2014.

13. Bergstra J, Bengio Y: **Random Search for Hyper-Parameter Optimization**. *J Mach Learn Res* 2012, **13**:281-305.

14. Claesen M, Simm J, Popovic D, Moreau Y, De Moor B: **Easy hyperparameter search using Optunity**. *arXiv preprint arXiv:14121114* 2014.

15. Ching T, Zhu X, Garmire LX: **Cox-nnet: An artificial neural network method for prognosis prediction of high-throughput omics data**. *Plos Computational Biology* 2018, **14**(4).
